# Mechanisms of microRNA export in mammalian cells: From random release to selective secretion

**DOI:** 10.1016/j.jbc.2026.113299

**Published:** 2026-06-26

**Authors:** Suvendra N. Bhattacharyya, Syamantak Ghosh, Sourav Hom Choudhury, Kamalika Mukherjee

**Affiliations:** 1 Department of Pharmacology and Experimental Neuroscience, University of Nebraska Medical Center, Omaha, USA; 2 Molecular Genetics Division, RNA Biology Research Laboratory, CSIR-Indian Institute of Chemical Biology, Kolkata, India; 3 Department of Anesthesiology, University of Nebraska Medical Center, Omaha, USA

**Keywords:** evolution of miRNA export, extracellular vesicles, gene expression regulation, intercellular communications, miRNA export, RNA binding proteins

## Abstract

Extracellular vesicles (EVs) are essential carriers of information between cells and tissues in multicellular (metazoan) organisms. MicroRNAs (miRNAs), key components of extracellular vesicles along with other non-RNA cargo, play a crucial role in regulating gene expression in both donor and recipient cells. Several questions about the basis and mechanisms of miRNA sorting into EVs have been raised, some of which have recently been addressed in reports on RNA-binding proteins and miRNA motifs that enable selective miRNA export. Recent studies indicate that both active and passive mechanisms of miRNA export occur in mammalian cells, underscoring that miRNA export is a selective and context-dependent process. This flexibility in the export process allows mammalian cells to respond quickly to changing environments. This review explores the mechanisms of miRNA export, the roles of RNA-binding proteins in this process, highlighting how these proteins enable the selective secretion of miRNAs across different mammalian cell types. EV-mediated export facilitates a targeted exchange mechanism for epigenetic signals like miRNAs, evolving from what initially appeared to be a random release process. Additionally, we have discussed the key unresolved questions regarding miRNA export and its evolution as a major mechanism of intercellular communication in metazoans.

Metazoan cells respond to their environment by rapidly switching the expression of genes that confer fitness for survival under challenging conditions (1). The exchange of genetic material allows recipients or donor cells to gain a selective advantage in combating these challenges (2). miRNAs are 17 to 23 nt long non-coding RNAs that serve as primary post-transcriptional regulators of genes in metazoan cells and contribute to translational repression and degradation of target mRNAs by base pairing and binding with imperfect complementarities. The Argonaute (Ago) family of proteins forms complexes with mature miRNAs and is indispensable for Ago-miRNP-mRNA interactions and miRNA-mediated gene repression (3, 4). The levels and activities of miRNAs in mammalian cells must be spatiotemporally regulated to ensure proper expression of their target genes (5). The diversity and abundance of miRNAs contribute to the epigenetic identity of cells, and this identity can be communicated or shared between cells *via* miRNA exchange across cell boundaries (6).

This exchange, including that mediated by extracellular vesicles loaded with specific miRNA sets, contributes to gene-expression homeostasis in metazoans (7, 8). Key unanswered questions concern how miRNAs are selected and loaded into vesicles for export, and which prerequisite factors influence miRNA selection. Identification of export motifs, RNA-binding proteins, and post-transcriptional modifications has become central to understanding miRNA export mechanisms (9). The development of *in vitro* assay systems has advanced mechanistic research on miRNA export processes (10). Understanding how miRNA export has evolved as a key mechanism of intercellular communication remains a compelling area of investigation. This review examines these issues and highlights how miRNA export regulation and evolution may have evolved across metazoan cells, from the non-specific, passive diffusion of RNA to an active, miRNA-selective export process.

Additionally, in this review, we have discussed questions related to the evolution of the miRNA export process, as well as the major challenges and obstacles associated with using miRNA-loaded extracellular vesicles (EVs) based therapy and their use as potent biomarkers. It has also explored how the EV-mediated miRNA exchange differs from other modes of intercellular signaling. The pathophysiological roles of EV-associated miRNAs and their preferential secretion by specific cell types are examined, despite the ubiquity of EV-mediated intercellular communication, to address key parameters and misconceptions regarding miRNA export and miRNA-mediated intercellular communication.

The existing literature includes an expanding body of studies exploring the mechanisms of miRNA export. However, there is a notable lack of a comprehensive review that outlines the entire process in metazoan cells, including the mechanisms involved, the regulatory proteins that act on them, and possible models that explain the detailed steps. In this review, we discuss these aspects of miRNA export, along with potential future research directions, to explore the commonalities and differences among the mechanisms reported.

## Transport of RNA across cell boundaries

### Active RNA-protein interaction or passive diffusion?

**Figure 1:**
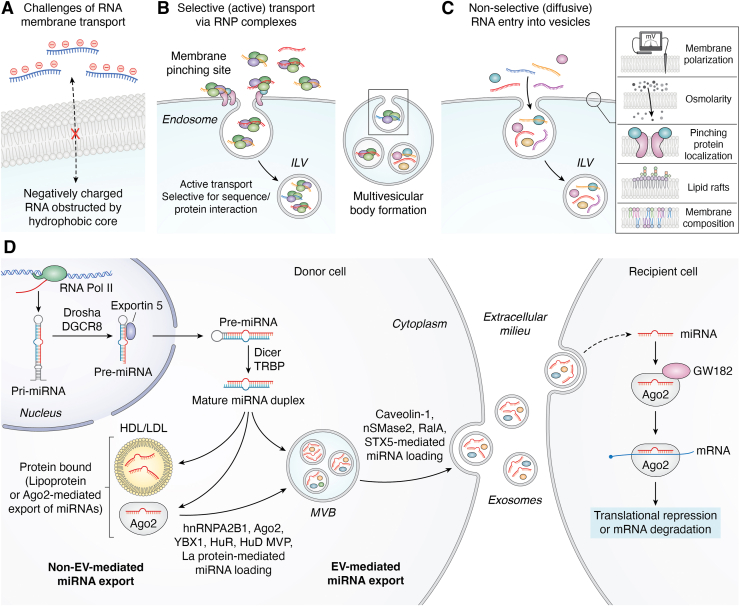
**miRNA transport across biological membranes.***A*, RNA, being a charged molecule, cannot traverse the hydrophobic core of the lipid membrane without assistance from other factors that can pass through it. *B*, the mechanism of membrane invagination and pinching facilitates the entrapment of RNA cargoes, in which RNA-protein complexes interact with membrane receptors that enable the selective entry of RNA into vesicular lumens. These vesicles are formed as part of the RNA-protein complex and are intended for subsequent trafficking to other destinations or fusion with another membrane, such as the plasma membrane. *C*, the non-selective, diffusion-driven entry of RNA into membrane-entrapped structures can account for the concentration-dependent uptake of RNA cargoes by vesicles. An annotation of key factors influencing these processes, including osmolarity, membrane polarization, lipid rafts, protein localization, and membrane composition, is provided. *D*, EV and non-EV pathways of miRNA export from mammalian cells. A schematic diagram shows the export of miRNAs *via* EVs from the donor cells and the uptake of those miRNAs by recipient cells. It also shows the various factors responsible for packaging miRNAs into MVBs for export *via* EVs. Other than EV-mediated pathways, miRNAs are also transported by lipoproteins, including both HDL and LDL. Moreover, it has also been reported that extracellular miRNAs can be found in the bodily fluids in the Ago2-bound state both in and out of membrane-derived vesicles.

RNA transport across biological membranes is restricted by the ionic and negatively charged nature of RNA, which makes it soluble yet difficult to traverse lipid bilayers (11) (Fig. 1*A*). The hydrophobic layer of the cell membrane makes it difficult for a charged ligand, such as RNA, to cross this barrier without binding to supporting molecules, such as RNA-binding proteins, that mediate transport by interacting with specific partner proteins, or by the presence of a channel permeable to large biomolecules, such as RNA–protein complex (12, 13). Therefore, for transport across the biomembrane, RNA should either adopt a strategy to traverse the lipid-rich domain or be retained within the cytosolic compartment and subsequently be entrapped by invaginating membrane structures to form vesicles, thereby allowing non-selective RNA entry into vesicles (14) (Fig. 1, *B* and *C*).

In the selective mode of RNA transport, RNA interacts with RNA-binding proteins to form RNP complexes that can traverse the biomembrane as the membrane pinches off with cytosolic content and forms vesicles within vesicles, as observed in the formation of multivesicular structures that facilitate subsequent transport within these vesicles, allowing the selective export of miRNAs (15) (Fig. 1*B*). Alternatively, the RNA–protein complex becomes part of a membrane structure pinched off directly from the plasma membrane. Interestingly, both processes require sufficient RNA-protein complexes at pinching sites and occur during membrane pinching, allowing RNA to participate in the pinching process and enabling secretion of the RNA-protein complex required for active transport rather than passive diffusion, thereby ensuring selectivity through RNA–protein and protein–protein interactions (16, 17). The apparent diffusive mode, or non-selective miRNA entry into vesicles, may also be affected by parameters such as osmolarity, membrane polarization, localization of proteins involved in membrane pinching, membrane lipid composition, or the presence of lipid rafts (18, 19) (Fig. 1*C*).

Depending on the sources and origins of the vesicles (cell membranes, endosomal membranes, or mitochondria-derived vesicles), the collective term extracellular vesicles is usually used as an umbrella term covering diverse membrane-entrapped cargo-containing structures (20). The Exosomes, or small extracellular vesicles with a more defined origin, cargoes, and protein markers, are of defined size and shape, and are considered a major component of extracellular vesicles or EVs (21, 22). In addition to microparticles and lipoproteins (18) or Ago complexes (23, 24), other cellular membrane-bound structures, such as microvesicles or MVs (also known as ectosomes), are directly derived from the plasma membrane and have miRNAs with them (25). Mitochondria-derived vesicles (MDVs), derived from mitochondria and carrying mitochondrial content (26), and apoptosomes (derived from apoptotic cell membranes with apoptotic markers secreted by dying cells) (27), are the other potential sources of miRNAs in the extracellular milieu. However, selectivity and a more defined mechanism for miRNA export *via* exosomes are well known, and we will focus our discussion on the exosomal miRNA export process, as these are of greater therapeutic and diagnostic value, and their importance to cell physiology and disease onset is more closely linked and better supported by highly cited literature (28, 29). The diverse types of extracellular vesicles, along with their characteristics and origins, are outlined in Table 1.

**Table 1:** Mode of miRNA export from animal cells

Export pathway	Sub-type/carrier	Key molecular mechanisms	Sorting & packaging machinery	Representative function & fate
Vesicular	Exosomes (Small EVs, ∼30–150 nm)	Endosomal membranes undergo inward budding to form Multivesicular Bodies (MVBs), which then fuse with the plasma membrane	• ESCRT-dependent or independent RNA-Binding Proteins (RBPs) like hnRNPA2B1, Ago2, and YBX-1, HuR/HuD • Ceramide-dependent pathways	Long-distance paracrine and endocrine signaling; uptake by target cells *via* fusion or endocytosis
	Microvesicles(Ectosomes), ∼100–1000 nm	Budding outward and direct fission of the plasma membrane	• Arf6, P4-ATPase • Cytoskeletal rearrangements • RBP-mediated tethering	Affecting local microenvironment and cell-to-matrix communication
	Apoptotic Bodies 1–5 μm	From blebbing and fragmentation of dying cells	• Caspase-dependent mechanisms • Passive non-selective packaging	Clearance by phagocytes; may contain degradation-resistant miRNAs
Non-Vesicular	Protein Complexes (*e.g.*, Ago2-miRNA)	miRNA associates directly with Argonaute proteins (mainly Ago2) or Nucleophosmin 1 (NPM1)	• Ago2 binds 3′ ends of miRNA • Post-translational modifications (*e.g.*, phosphorylation) regulate release	Protects miRNA from RNase degradation in circulation; delivers miRNA to specific receptors
	Lipoprotein Complexes (*e.g.*, HDL/LDL)	miRNAs bind to High-Density or Low-Density Lipoproteins	• Ceramide pathway-dependent export • Interaction with membrane transporters	Delivers miRNAs to distant tissues utilizing established lipid transport and receptor pathways (*e.g.*, SR-BI)
	Free/Unbound	Minor fraction of actively secreted unbound miRNA	• Energy-dependent active export pulse mechanisms	Paracrine signaling; largely susceptible to extracellular RNase degradation without protective complexes

Interestingly, RNA exchange between cells occurs not only in eukaryotes but also in prokaryotic communities (30). In bacteria, RNA transfer occurs *via* membrane vesicles, including outer membrane vesicles (OMVs) in Gram-negative bacteria and cytoplasmic membrane vesicles (CMVs) in Gram-positive bacteria, similar to how human cells use exosomes (31). This mechanism enhances horizontal gene transfer and genetic diversity within bacterial populations, aiding their adaptation and survival in changing environments (32). RNA transport across membranes occurs in lower eukaryotes; for instance, nuclear-encoded cytoplasmic tRNAs (transfer RNAs) are imported into mitochondria in unicellular eukaryotic organisms such as *Leishmania* and *Trypanosoma* (33, 34). Mitochondrial tRNA import also occurs in yeasts, plants, and marsupials (34). Additionally, chloroplasts import specific nuclear-encoded tRNAs to support translation and other functions within the organelle (35).

In eukaryotic cells, the transport of mRNAs and other RNAs, such as rRNAs (ribosomal RNAs) and tRNAs, out of the nucleus has been extensively studied, and their roles in gene regulation have been documented (36). miRNA precursors (Pre-miRNAs) are exported from the nucleus by Exportin 5-RAN GTPase (37). The fundamental mechanism of RNA transport across biomembranes, observed in prokaryotes and lower eukaryotes, is either adapted or fully recapitulated in higher metazoan animals for the transfer of miRNA and other RNAs, suggesting a universal biophysical and biochemical principle of RNA transport that has persisted throughout evolution.

### miRNA homeostasis and transport processes

Mature miRNAs are only 17 to 23 nucleotides long and predominantly exist as part of the Ago miRISC (miRNA-induced silencing complex), which is essential for miRNA-mediated repression and also as a substrate for miRNA deactivation either by Ago phosphorylation (38), miRNA uncoupling of Ago (39), and uridylation of miRNA for degradation (40). miRNAs are also found associated with the outer membranes of various organelles, including the endoplasmic reticulum, endosomes, lysosomes, and RNA processing bodies (P-bodies), possibly *via* RNA-binding proteins (41, 42, 43). Additionally, miRNAs are associated with mitochondria and lipid droplets (44, 45).

Studies have shown that their subcellular localization affects miRNA-mediated gene repression, especially under conditions that alter their distribution (46). The import of miRNAs into double-membraned mitochondria and single-membraned endosomes has important implications for cellular metabolism and apoptosis (44). Mitochondrial miRNAs can also be targeted to mitovesicles, a special class of extracellular vesicles that carry mitochondrial content out of the cell, either *via* endosomal pathways or independently (26). Another crucial method of RNA transfer involves nanotubes, which are slender, membrane-enclosed structures that connect cells over both short and long distances (47). These tunneling nanotubes (TNTs) facilitate the direct exchange of cellular contents, including RNA molecules, between connected cells. TNTs play a vital role in various normal and pathological processes, such as immune responses, cancer progression, and tissue regeneration (48). For instance, the transfer of mRNAs and lncRNAs (long non-coding RNAs) *via* TNTs can alter gene expression patterns in recipient cells, trigger adaptive responses, or contribute to disease development (49).

Additionally, miRNAs are transported by RNA-binding protein complexes that protect them from degradation and facilitate their passage across the cell membrane. For example, high-density lipoproteins (HDLs) serve as carriers for miRNAs in the bloodstream, maintaining their stability and facilitating delivery to target cells, where they modulate gene expression. This highlights the interplay between lipid metabolism and RNA-based regulation of cellular functions (50). Furthermore, RNA-binding proteins (RNA-binding proteins) guide RNAs to specific destinations within the cell, including extracellular vesicles and nanotubes, ensuring targeted delivery. This process underscores the essential role of RNA-binding proteins in the selective packaging and transport of RNA molecules (12) (Fig. 1*D*).

### Vesicles that carry miRNAs: cargo specificity vs diversity

The detection of small vesicles near healthy mammalian cells in electron micrographs or body fluids was first reported in the late 1960s (51, 52). The term “extracellular vesicles” (EVs) was only introduced around 2011 to describe all lipid bilayer-enclosed extracellular structures (53). Initially, it was thought that cells released EVs to remove surplus proteins, but late-1990s studies suggested that EVs could facilitate intercellular communication, especially in immune responses and cancer (54, 55). In 2007, it was shown that EVs contain functional mRNAs and miRNAs that can modulate recipient cell behavior upon uptake (56). Subsequently, researchers found that non-exosomal EVs, such as microvesicles, microparticles, and ectosomes, originating from the plasma membrane, could transfer functional proteins and RNAs between cells (57).

**Figure 2:**
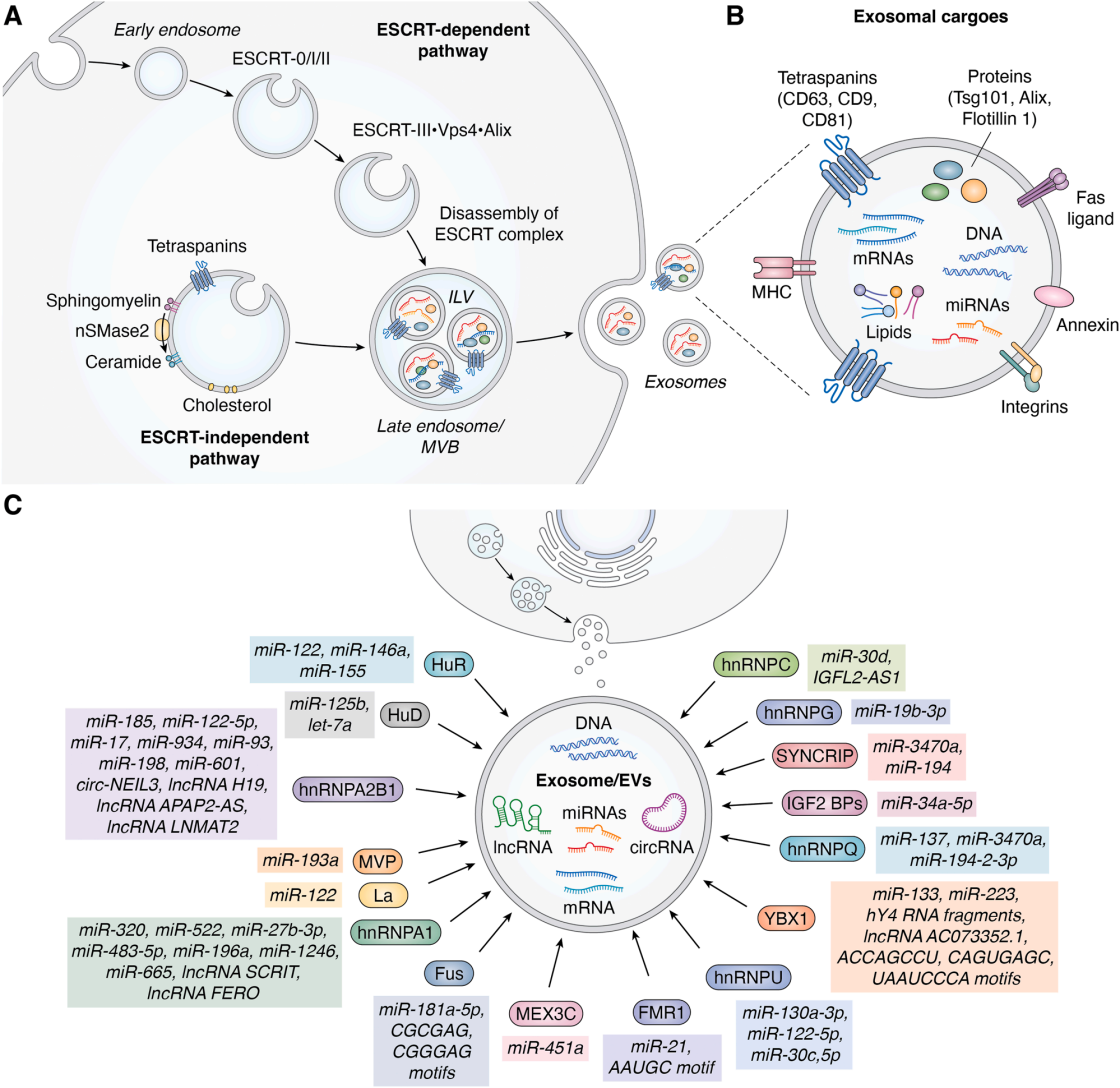
**Classes of cargoes exported *via* EVs with possible export pathways and involvement of RNA-binding proteins.***A*, schematic diagram showing multivesicular body (MVB) biogenesis and export *via* both ESCRT-dependent and ESCRT-independent pathways. *B*, simplified illustration showing different cargo molecules packaged inside the EVs. *C*, classes of RNA-binding Proteins (RBPs) and their cargos for EV-mediated export. A broad spectrum of RBPs, including hnRNP family members, HuR, YBX1, IGF2BPs, and SYNCRIP, among others, is involved in recruiting RNA into EVs. Various colored solid shapes represent different RNA-binding proteins; rounded rectangles of matching colors represent the miRNAs and lncRNAs sorted by these RNA-binding proteins. The double-layered membrane structure at the center symbolizes exosomes (EVs) loaded with assorted cargo. A simplified diagram at the *top* centre illustrates the biogenesis of EVs/exosomes. Key abbreviations: EVs, extracellular vesicles; FMR1, fragile X messenger ribonucleoprotein 1; FUS, fused in sarcoma; hnRNP, heterogeneous nuclear ribonucleoprotein; HuD, Human antigen D; HuR, human antigen R; IGF2BPs, insulin-like growth factor 2 mRNA-binding proteins; La, Lupus La protein; LncRNA, long non-coding RNA; MEX3C, muscle excess 3 RNA binding family member C; MVP, major vault protein; RBPs, RNA binding proteins; SYNCRIP, Synaptotagmin-binding cytoplasmic RNA-interacting protein; YBX1, Y-box binding protein 1.

The export process for each EV type involves unique mechanisms. Small extracellular vesicles(sEV) or Exosomes form through endosome maturation into multivesicular bodies or MVBs, which originate from inward budding of the endosomal membrane, producing intraluminal vesicles that mature into exosomes or sEVs (Figs. 1*D* and 2*A*). The ESCRT (endosomal sorting complexes required for transport) machinery regulates this process, though ESCRT-independent paths involving tetraspanins and lipid rafts also exist. Mature MVBs fuse with the plasma membrane in a Rab GTPase-dependent manner, particularly involving Rab27a and Rab27b, thereby releasing exosomes into the extracellular space (58). Microvesicles or MVs form *via* outward budding and fission of the plasma membrane, regulated by cytoskeletal components such as actin and myosin, and by lipid rearrangements, including phosphatidylserine translocation from the inner to the outer leaflet, the two separate, single layers of phospholipids, proteins, and lipids that form a biological lipid bilayer. Small GTPases like ARF6 and RhoA coordinate membrane dynamics for vesicle budding (59). Apoptotic bodies are produced during apoptosis and are characterized by cell shrinkage, chromatin condensation, and membrane blebbing. Caspases degrade cellular components, forming apoptotic bodies that contain various cellular components, including miRNAs (60). Particles smaller than 50 nm, once thought to define exosomes, are now mainly recognized as non-vesicular nanoparticles lacking lipid bilayers. These include exomeres (35–45 nm), characterized by unique proteomic profiles and enrichment in metabolic enzymes (61), and supermeres (22–32 nm), which are dense in extracellular RNA and carry disease biomarkers, making them highly efficient for cellular uptake (62). Their origin remains debated; they may be actively secreted or simply breakdown products passively released by cells. Because the size is below the detection limit of standard nanoparticle tracking analysis (70–90 nm), advanced techniques such as Flow Field-Flow Fractionation (AF4) and atomic force microscopy (AFM) are required for the study (63).

The selective packaging of miRNAs into EVs affects their functional and communicative roles. Exosomes, derived from endosomes, often deliver miRNAs that regulate gene expression, thereby affecting immune responses, tumor growth, and tissue repair (29, 64, 65, 66). Microvesicles (MV), larger and released directly from the membrane, encapsulate diverse miRNAs involved in stress and inflammation responses, playing a role in rapid signaling and reactions to stimuli. MV miRNA profiles can reflect cellular activation status and influence inflammation, immunity, and tissue repair (53, 67). Apoptotic bodies, generated in cell death, deliver miRNAs that modulate apoptotic cell clearance and inflammation resolution. These miRNAs can be taken up by phagocytes, such as macrophages, thereby influencing their behavior and promoting anti-inflammatory processes that support tissue homeostasis and regeneration (68). Overall, miRNA transport *via* EVs is mediated by tightly regulated mechanisms that facilitate specific intercellular communication. Each EV type employs distinct packaging and release pathways, shaped by cellular signals and molecular interactions, ensuring that miRNA cargo aligns with the cell’s functional state, whether in health or disease. Studying these pathways enhances understanding of EV functions and highlights potential therapeutic targets for regulating cell communication (Figs. 1*D* and 2*A*; Table 1).

### miRNA export control in diseases: EV-associated miRNAs as biomarkers and potential therapeutic agents

The majority of cells from various tissues, whether normal or diseased, release EVs containing a range of miRNAs and other factors that play crucial roles in regulating disease pathophysiology (69). These EV-associated miRNAs can be secreted by affected and diseased cells and subsequently taken up by neighboring cells, where they influence gene expression and cellular behavior, including inflammatory responses, cell migration, proliferation, apoptosis, autophagy, angiogenesis, and epithelial–mesenchymal transition (70). Several miRNAs, such as miR-7, miR-92a, miR-9, miR-21, miR-29c, miR-32, miR-34a, and miR-125b, regulate key physiological pathways in various cancers, including lung, colorectal, breast, gastric, and liver cancers, as well as melanoma (71, 72). It has been observed that EV-associated miR-21 and miR-29a can activate the NF-κB pathway and promote the secretion of pro-metastatic inflammatory cytokines by binding to toll-like receptors in immune cells, ultimately contributing to tumor growth and metastasis (73, 74). Conversely, miR-146 and let-7a function as tumor-suppressor miRNAs released *via* EVs by different cancer cell types, thereby regulating tumor progression (75, 76). The diverse actions of EV-miRNAs across various diseases and pathological conditions are increasingly documented in scientific literature.

Studies have also demonstrated that, beyond cancer, extracellular miRNAs significantly contribute to the development of cardiovascular diseases such as atherosclerosis, heart failure, acute coronary syndrome, myocardial ischemia, and pulmonary hypertension. Let-7a, miR-155, miR-21, miR-146a, miR-92a, and miR-181a exhibit differential expression profiles in patients with cardiovascular diseases, as they modulate critical cellular pathways that reduce atherosclerosis, control inflammation, and enhance myocardial function (77).

Disruption in the regulation and altered expression of various cellular and extracellular miRNAs have been associated with neurodegenerative diseases like Alzheimer’s disease, Parkinson’s disease, amyotrophic lateral sclerosis, and multiple sclerosis. EV-associated miR-21-5p, miR-124-3p, among others, have been identified in cerebrospinal fluid (CSF), serum, and plasma samples from Alzheimer’s patients, exhibiting altered expression patterns (78, 79). Similarly, in patients with Parkinson’s disease, serum- or CSF-associated EV miRNAs, such as miR-7 and miR-133b, show differential expression compared with healthy controls (80, 81) (Table 2).

**Table 2:** EV-Associated miRNAs and target genes

Disease	EV-associated miRNA	Target gene(s)	Function/Effect
Alzheimer's disease (AD)	miR-375	APH1B, CELF2	APH1B is part of the γ-secretase complex involved in APP cleavage (258)
	miR-107	CDC42SE2, IL6	Modulates actin cytoskeleton and inflammation (259).
	miR-193b	APP	Regulates amyloid precursor protein levels (260).
	miR-29c	BACE1, DNMT3	BACE1 is involved in Aβ generation (261).
	miR-613	BDNF	Regulates neuronal survival and differentiation (262).
	miR-146a	TLR2	Mediates innate immune response and neuroinflammation (263).
	miR-128	PPARG	Involved in lipid/glucose metabolism and Aβ clearance (264).
	miR-206	IGF1	Linked to neuroprotection and inflammation (265).
Coronary artery disease	miR-126	Smooth muscle genes	Promotes proatherogenic phenotype in smooth muscle cells (266).
	miR-320a	IGF-1 signaling	Impairs angiogenesis and vascular remodeling (267).
Hepatocellular carcinoma	miR-183-5p, miR-19a-3p	Multiple metabolic genes	Panel of 5 miRNAs used as diagnostic biomarkers (268).
	miR-224	Not specified	Diagnostic and prognostic marker (269).
Ischemic stroke	miR-18a-5p, miR-199a-5p	HIF1A, ANKRD12, GNAI2	Regulates response to ischemia (270).
Lung adenocarcinoma	miR-10b	Not specified	High-accuracy diagnostic biomarker (271).
Esophageal cancer	miR-130a-3p	METTL14/FSP1	Confers cisplatin resistance by regulating ferroptosis (272).
Sepsis-induced ARDS	miR-511-5p, miR-150-5p, miR-193b-5p	TLR4, IL6, TP53, STAT1	Central roles in lung injury signaling (273).
Focal cortical dysplasia	Multiple (8 miRNAs)	CDKN1A, CD274	Enriched in immune and p53 signaling pathways (274).

The process of translating extracellular vesicle (EV)-associated microRNAs (miRNAs) into clinical liquid biopsies encounters significant technical, biological, and analytical challenges. Despite their structural stability and ability to reflect tissue conditions, current protocols lack the precision necessary for routine diagnostic use. Key issues include the trade-off between purity and yield in isolation methods—such as ultracentrifugation being time-consuming and risk of EV aggregation, *versus* rapid precipitation kits that often contaminate samples with proteins and free miRNAs. Additionally, co-isolation of contaminants like high-density lipoproteins complicates miRNA profiling, while the extremely low abundance of target miRNAs per vesicle demands highly sensitive extraction techniques (82). Biological complexities further hinder progress: multiple EV subtypes are secreted simultaneously, yet current tools cannot efficiently distinguish disease-specific miRNAs among these subpopulations (83).

Moreover, systemic factors such as age, metabolic syndrome, and inflammation influence circulating miRNA levels, creating background noise that complicates specificity assessment. Variations in EV profiles across body fluids add another layer of difficulty, as samples such as blood plasma, urine, and saliva exhibit markedly distinct miRNA profiles even within the same patient (84). Methodologically, there’s a lack of standardized internal controls for normalization in EV-qPCR assays, risking misinterpretation. Pre-analytical variables—including storage conditions and handling procedures—also impact vesicle integrity and data reliability. Most studies are limited by small, single-center cohorts and lack the comprehensive validation necessary for clinical approval (85). Overall, these multifaceted barriers hinder the reliable incorporation of EV-associated miRNAs into routine liquid biopsy diagnostics.

### Preferences in EV secretion

Specific cell types with predominance in EV Secretion are reported. While nearly all living cells, from prokaryotes to eukaryotes, can secrete extracellular vesicles (EVs), some are more "prolific" or favored in research and clinical applications due to their abundance, safety, or therapeutic potential (86). Certain cells are particularly known for producing high yields or specialized EVs (87). Blood plasma is a common source of EVs because of its abundance. Platelets are the primary contributors to EVs in healthy blood and increase in number during inflammation or illness (88, 89). Red blood cells (RBCs) are well-suited for large-scale production, such as for drug delivery, as they lack nuclear and mitochondrial DNA, thereby reducing gene-transfer risks (69, 90). Cancer cells release EVs prolifically to modify their environment and support metastasis (91). Mesenchymal Stem Cells (MSCs) are valuable in regenerative medicine because their EVs carry signals for angiogenesis and repair without the risks associated with stem cell transplantation (92). Some tissues, like adipocytes (fat cells), skeletal muscle, and cardiomyocytes (heart muscle cells), produce fewer EVs and are considered "conservative" EV producers (93, 94). The rate and type of EV secretion can vary depending on environmental factors. Activated immune cells, such as mature dendritic cells or T cells, tend to secrete more and different EVs than their immature counterparts (95). Conditions such as hypoxia (low oxygen), acidity, and cellular damage significantly enhance EV production, especially in tumor environments (96). The secretion mechanism also varies: some cells prefer to bud directly from the plasma membrane (microvesicles or MVs/Ectosomes), while others use the endosomal pathway (exosomes or sEVs).

All these EV-associated miRNAs exhibiting abnormal expression in the bodily fluids of individuals with various disease conditions reflect underlying pathophysiological processes and could be important for creating diagnostic biomarkers for clinical application. Despite the clinical potential of using exosomal miRNAs as therapeutic agents, several technical and scientific challenges remain. A major limitation of using EVs in clinical applications is the availability of pure EVs from the body. All the available EV isolation techniques have their own advantages and disadvantages. Bodily fluids are often considered the reservoir of EV and EV-associated miRNAs, but these are released from both disease-affected and normal cells.

### Linking specific EV-miRNAs to diseases

Identifying which miRNAs are specifically linked to the disease conditions is an essential prerequisite before proceeding with the biomarker study. Moreover, miRNA biomarkers are largely nonspecific, associated with a wide range of conditions and outcomes. Even in similar studies of patients with the same disease, there has been very limited overlap in findings (97). Additionally, some of these EV-associated miRNAs regulate other key cellular pathways; therefore, targeting them therapeutically might affect normal physiological processes in an individual (Tables 2 and 3). So, the question remains: will it be possible to reach the next level and move miRNA biomarkers from theory to clinical reality?

**Table 3:** EV-associated miRNA biomarkers

miRNA	Associated condition	Clinical Use/Role	Source fluid
miR-21/miR-21-5p	Glioblastoma, HCC, Melanoma, NSCLC (275)	Diagnostic/Chemoresistance	Serum, Plasma
miR-122/miR-122-5p	Hepatocellular Carcinoma (HCC), Aging (276)	Diagnostic (Downregulated in HCC)	Serum, Plasma
miR-150-5p	Neuroblastoma, NSCLC, Breast cancer (277, 278)	Diagnosis/Risk Stratification	Plasma
miR-375	Prostate cancer, Alzheimer’s (AD) (279)	Predictive (Castrate-resistant)/Diagnostic	Blood, Serum
miR-126-3p/126-5p	Melanoma, Chronic kidney disease (CKD) (280)	Metastatic disease/Risk scoring	Serum, Plasma
miR-193b-5p/193a-5p	Carotid artery stenosis, Oral cancer (281)	Plaque detection/Early diagnosis	Plasma, Saliva
miR-205-5p	Cancer-related stroke (282)	Predicting coagulopathies	Plasma
miR-1290	Prostate Cancer, Pancreatic cancer (283)	Castrate-resistance/High accuracy	Blood
miR-223-3p	NAFLD, NSCLC (284)	Diagnostic (Elevated in NAFLD)	Plasma
let-7d-5p	Chronic kidney disease (CKD), NSCLC (285)	Predictive of advanced CKD	Plasma
miR-142-5p	Triple-negative breast cancer (TNBC) (286)	Diagnostic	Serum, Plasma
miR-199a-3p	Parkinson's disease (PD), HCC (287)	Early diagnosis/Progression	Serum

MiRNAs linked to extracellular vesicles are highly effective biomarkers for diagnosing, monitoring, and predicting disease progression. Their main clinical advantage is the lipid bilayer of EVs, which shields enclosed miRNAs from enzymatic degradation in body fluids such as blood and urine, offering greater stability than cell-free miRNAs. EV-miRNAs’ advantages include stability from the EV membrane against degradation, detectability after multiple freeze-thaw cycles, selective packaging for higher sensitivity and specificity, and ease of collection *via* minimally invasive liquid biopsies using serum, plasma, saliva, or urine. Challenges in clinical use include inconsistent EV isolation methods, variability in EV size and cargo, and the need for advanced technologies such as next-generation sequencing (NGS) and qRT-PCR for profiling (98, 99). The need for standard operating procedures that ensure the isolation and quantification of human-error-free (automation) will enable large-scale batch processing of multiple clinical samples with high reproducibility of results and consistent performance across different experimental settings (100, 101). To that end, several methods and techniques have recently received regulatory approval for use as diagnostic kits for EV-miRNA-based detection of cancer or neurodegenerative diseases (102).

### Engineered EVs as drug delivery vehicles

Recent studies highlight EVs' role in drug delivery, as they carry proteins, lipids, and nucleic acids and evade the immune response due to their small size and similarity to parental cells. Their paracrine effects and circulatory retention enhance clinical relevance (103). However, future researchers should adopt a combinatorial approach that integrates multiple methods, including ultracentrifugation, antibody-based isolation, FACS, Western blotting, nanoparticle tracking, and electron microscopy, to optimize the isolation and characterization of EVs and their biophysical and biochemical properties. Future researchers should employ a range of methods to optimize EV isolation and characterization. Reports indicate that removing EVs from circulation or the uptake of faulty EVs reduces tumorigenesis. EVs can also be isolated from a patient's blood, modified, and returned for cancer therapy (72, 104). Tumor liquid biopsy could be key in addressing specificity in EV therapeutics. It's a highly specific, sensitive, non-invasive, and reproducible method for real-time patient monitoring. Additionally, it accurately detects altered levels of EV and EV-associated miRNAs (105, 106). Although EV-associated miRNAs can be excellent diagnostic and therapeutic tools for several diseases, their translation into clinical practice still requires ongoing research with high reproducibility. Technical advances to isolate and explore EVs at the single-particle level are essential for addressing several questions about EV heterogeneity and uniqueness in body fluids.

It is interesting that the significant clinical potential of using modified EVs as therapy or biomarkers is limited by the lack of mechanistic understanding of how miRNAs are selected for export. This includes the basis for cargo selection and the cellular and pathogenic factors that control this process. Currently, understanding miRNA export requires additional research and resources to develop engineered EVs that carry specific miRNAs and other gene-modulatory agents to modulate pathophysiological conditions and to treat various human diseases.

Thus, engineered EVs serve as nanocarriers meticulously designed for the targeted delivery of therapeutic agents to specific cells. Naturally occurring EVs facilitate intercellular communication by transporting proteins and nucleic acids; however, engineering advancements have significantly enhanced their capacity for drug loading and tissue specificity (107, 108). Modification techniques encompass surface decoration with ligands, antibodies, or peptides, such as the RVG peptide, which enables crossing the blood-brain barrier for neurodegenerative conditions (109). Cargo-loading methodologies include endogenous approaches, such as genetically engineering donor cells, as well as exogenous techniques, such as electroporation, sonication, or freeze-thaw cycles (110, 111). Cell engineering applications include improving EV stability, reducing immunogenicity, or increasing production yields (112). Compared with synthetic lipid nanoparticles, EVs are less likely to be perceived as foreign, demonstrate superior fusion with target cells, and can traverse barriers such as the blood-brain barrier and the tumor microenvironment. Their applications in medicine include delivering anti-tumor agents, siRNAs, neurotrophic factors, and anti-HIV compounds to treat various cancers, neurological disorders, and infections (110, 113, 114).

Engineering extracellular vesicles (EVs) holds significant potential for targeted drug delivery and diagnostic applications, but numerous technical and biological challenges must be addressed before they can be widely adopted in clinical practice (115). Production and isolation yield low amounts from natural cells; scalability issues with current methods; difficulty separating EVs from similar particles such as retroviruses and protein aggregates; and a lack of standardized protocols, leading to batch variability (116). Characterization is hindered by their nanoscale size, heterogeneity in cargo and size despite originating from the same cell line, and limitations in accurately quantifying cargo loading (117). Cargo loading methods are often inefficient: passive incubation yields low drug encapsulation, active techniques such as electroporation or sonication risk membrane damage or aggregation, and endogenous loading *via* cell transfection results in low sorting efficiency (118). Biological and targeting obstacles include rapid clearance by the liver and spleen, insufficient tissue-specific targeting, endosomal trapping of EVs, which leads to cargo degradation, and immune responses due to donor-recipient mismatch (119). Regulatory and storage issues include difficulties in classifying EV therapies, their instability, and a tendency to degrade or aggregate without costly ultra-low-temperature storage (120). Addressing these challenges may involve innovative solutions such as bioreactors, synthetic hybrid EVs, or improved targeting and stability strategies (121).

### Mechanism of export: RNA-binding proteins and interacting partners regulating miRNA export

All potential uses of EV-miRNA described in the previous section rely on a crucial step: understanding how miRNA cargoes are incorporated into EVs. miRNA export involves protein interactions that either change the subcellular location of the target RNA or induce its post-transcriptional modifications. These modifications enable the selective entry of altered RNAs into endosomes through recognition by specific RNA interactors (122). Alternatively, RNA can form a complex with a specific protein, facilitating its transfer into the endosomal lumen and subsequent export *via* exosomes. Research on miRNA transport has mainly focused on identifying proteins involved in RNA-protein interactions, thereby uncovering key miRNA interactors necessary for export. This overview explains the mechanisms governing this process, highlighting how certain RNA sequences, motifs, or interactors, by engaging specific miRNA sets, drive their selective export (Fig. 2, *A* and *B*, Table 4).

**Table 4:** List of RNA-binding and non-RNA-binding factors controlling miRNA export

Name of the protein	Mechanism	Downstream function of regulated miRNAs
Exosome pathway/RNA-binding proteins		
Heterogeneous nuclear ribonucleoprotein A2B1 (hnRNPA2B1)	Promotes the EV-mediated export of miR-198 and miR-601 *via* potential binding with GGAG/UGCA motifs (130)	miR-198 loaded into EVs *via* a p62-mediated, autophagy-related pathway, act as a tumor suppressor in certain cancers by targeting genes like MIB1 (288).
Heterogeneous nuclear ribonucleoprotein A2B1 (hnRNPA2B1)	Promotes EV-mediated export of miR-17 and miR-93 *via* potential binding with AGG/UAG motifs (132)	EV-containing miR-17/93 as regulators of macrophage activation and as prognostic indicator of cancer and stoke (289)
Heterogeneous nuclear ribonucleoprotein C1 (hnRNPC1)	Promotes EV-mediated export of miR-30d during early development (136)	miR-30d increases the expression of genes essential for embryonic adhesion and invasion (such as Itgb3, Itga7, and Cdh5), thereby improving implantation rates (290)
Synaptotagmin-binding cytoplasmic RNA-interaction protein (hnRNPQ1)	Promotes EV-mediated export of miR-3470a and miR-194-2-3p *via* potential binding with GGCU motif *via* NURR domain (137)	miR-3470a is implicated in liver regeneration, while miR-194-2-3p has been associated with hepatocellular carcinoma (HCC) (291)
Y-Box Binding protein 1 (YBX1)	Promotes EV-mediated export of miR-133 and miR-223	miR-133 in exosomes aids in heart function and, along with other miRNAs, can promote muscle recovery (292).miR-223 delivered by macrophage-derived EVs can inhibit tumor proliferation in hepatocellular carcinoma (293)
Human antigen (HuR)	Binds miR-122, let-7a and miR-21 *via* RNA recognition motif III and promotes their loading into MVBs for subsequent export (141, 144, 205)	with miR-21 typically acting as an oncogene (upregulated) and let-7a often acting as a tumor suppressor (downregulated), while EV-miR-122 can modulate metabolic environments to aid metastasis (294, 295)
Human antigen D (HuD)	Binds let-7a and miR-125b in neuronal cells and loading into MVBs for subsequent export (146)	let-7a and miR-125b are key microRNAs that co-regulate neuronal cell fate, differentiation, and maturation, often working together to promote the transition from neural progenitors to mature neurons (296)
Argonaute 2 (Ago2)	Part of RISC; Phosphorylation-dependent sorting of miRNAs like let-7a, miR-100, miR-320a to EVs (196)	let-7a, miR-100, and miR-320a are microRNAs (miRNAs) acting as tumor suppressors, regulating cell differentiation, and controlling apoptosis, with high conservation across species (297)
La protein (La)	Promotes EV-mediated export of miR-122 in breast cancer cells (158)	Breast cancer (BC) cells utilize extracellular vesicles (EVs) to export miR-122, a microRNA that acts as a systemic "soil conditioner" for metastasis (298)
Major Vault Protein (MVP)	Binds and exports miRNA like miR-193a (163)	(EV)-associated miR-193a functions as a key mediator in tumor progression and intercellular communication (163)
Poly(rC)-binding protein 2 (PCBP2)	Impairs SYNCRIP-mediated loading of CELL-embedding miRNAs like miR-3470a and miR-194-2-3p (299)	miR-3470a is implicated in liver regeneration, while miR-194-2-3p has been associated with hepatocellular carcinoma (HCC) (291)
Mex-3 RNA Binding Family Member C (MEX3C)	Acts as an RNA-binding E3 ubiquitin ligase that works with Ago2 to sort miR-451a into exosomes (300)	enriched in serum exosomes, regulates macrophage and dendritic cell immune responses, and can be engineered into EVs to induce apoptosis in cancer cells, such as hepatocellular carcinoma (301)
Fragile X Messenger Ribonucleoprotein 1 (FMR1)	Binds AAUGC motif; inflamasome-mediated (302)	During inflammation, inflammasome-mediated signaling triggers FMR1 to load miRNAs like miR-155 into extracellular vesicles (EVs/exosomes) *via* the ESCRT machinery (302)
Aly/REF export factor (ALYREF) and Fused in Sarcoma (FUS)	Binds CGGGAG motif	They are known to regulate the export of specific miRNAs, such as miR-483-5p and miR-3622-5p, which contain this motif (9)
Exosome Pathways/Non-RNA-binding proteins		
Caveolin-1	Promotes sorting of hnRNPA2B1 and hnRNPA2B1-associated miRNAs into MVBs for export under stress conditions (133)	miR-198 loaded into EVs *via* a p62-mediated, autophagy-related pathway, act as a tumor suppressor in certain cancers by targeting genes like MIB1 (288)
Neutral Sphingomyelinase 2 and Annexin A2 (ANXA2)	Promotes sorting of miR-16 into EVs in tumor cells (168, 303)	Disposition of tumor-suppressive miRNAs like miR-16. By shuttling miR-16 into EVs for export, the cancer cell can reduce its intracellular levels of this miRNA, thereby preventing apoptosis and promoting proliferation. once released, these EVs can also transfer miR-16 to surrounding cells in the tumor microenvironment to modulate signaling (304)
Vacuolar Protein Sorting-Associated Protein 4 (VPS4)	Promotes EV-mediated export of oncomiRs including miR-27b-3p and miR-92a-3p in hepatocellular carcinoma cells (169)	The EV-mediated export of these oncomiRs, particularly miR-92a-3p, is heightened in highly metastatic HCC cells. These secreted EVs (containing miR-92a-3p) facilitate cancer progression by promoting epithelial-to-mesenchymal transition (EMT), angiogenesis, and metastasis (305)
Ras-like small GTPase, RalA	Promotes EV-mediated export of miR-122, miR-146a and let-7a in association with HuR using GTPase activity (144)	Exported miR-122 and miR-146a manage cellular responses to stress and inflammation, with miR-146a specifically reducing inflammation by inhibiting the NF-*k*B pathway (142, 144)
Syntaxin 5 (STX5)	Promotes EV secretion and EV-mediated export of miR-122 and let-7a *via* potential binding with miRNAs and HuR (145)	Exported miR-146a manage cellular responses to stress and inflammation, with miR-146a specifically reducing inflammation by inhibiting the NF-*k*B pathway (144, 145)
Caspase1/RILP	Caspase-1 dependent cleavage of RILP; moves MVB to periphery (306)	Various miRNAs and targets
Non-exosomal pathways (Direct-EV Targeting)		
Connexin43 (Cx43)	Forms channels at EV surface; binds miRNAs directly like miR-133b (307)	Cx43-mediated sorting of miR-133b into EVs has been shown to modulate autophagy flux in recipient cells (307)
Heterogeneous nuclear ribonucleoprotein (hnRNPU)	Regulates sorting into large EVs (307)	hnRNPU downregulation increases miR export into large EVs, which can modulate the migratory capacity of uptake (307)

### Balancing Ago-miRNA level and ESCRT-dependent export of EXOmotif-containing RNAs

**Figure 3:**
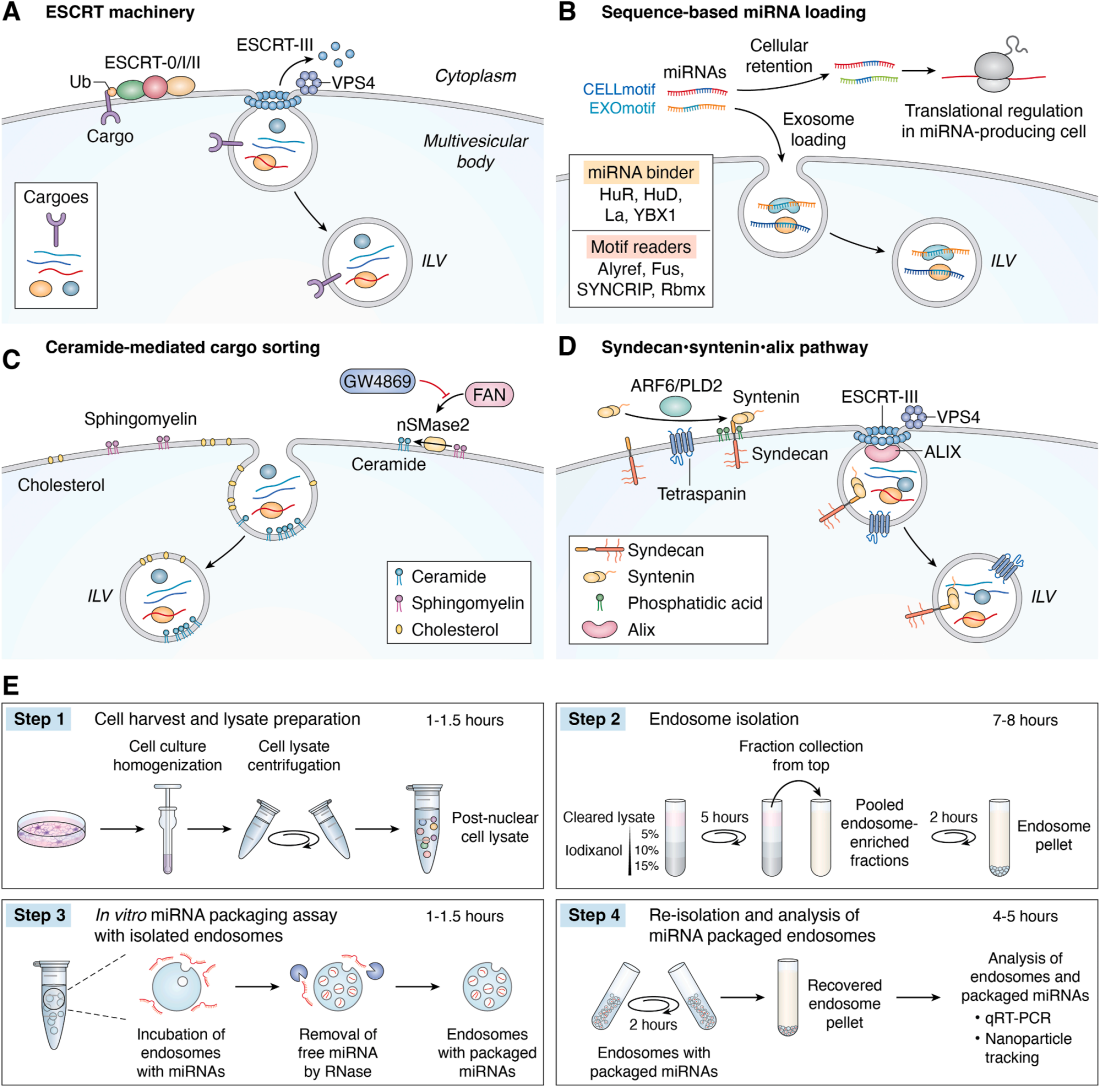
**Pathways and mechanisms of miRNA cargo selectivity.** Several molecular mechanisms for sorting miRNA and non-coding RNA into the intraluminal vesicles (ILVs) of multivesicular bodies (MVBs) have been identified: *A*, Tthe classical ESCRT-dependent pathway involves recognition of ubiquitinated proteins at the endosomal membrane by the ESCRT-0, -I, -II, and -III subcomplexes. The ATPase VPS4 mediates ILV formation in a stepwise manner. *B*, the sorting and cellular retention of miRNA into exosomes are mediated by cell-type-specific arrays of EXOmotifs and CELLmotifs. Reader proteins such as Alyref and Fus recognize these motifs on miRNAs, facilitating the incorporation of EXOmotif-containing miRNAs into exosomes. RNA-binding proteins such as HuR or YBX1 that may recognize non-EXOmotif sequences in miRNAs for export are classified separately. *C*, ceramide, generated from sphingomyelin by nSMase2, plays a crucial role in the ESCRT-independent pathway of ILV biogenesis. Ceramide can form lipid raft microdomains and induce the conversion of ILVs into MVBs. Activation of nSMase2 by FAN can be pharmacologically inhibited by small molecules such as GW4869. Cargoes sorted *via* this pathway include flotillin, cholesterol, and tetraspanins, which are localized in lipid rafts. *D*, in the syndecan-syntenin-ALIX pathway, membrane budding and cargo clustering can occur independently of the early ESCRT machinery, with VPS4 required for the scission step. *E*, *Iin vitro* endosome miRNA targeting assay. The stepwise isolation of endosomes, incubation with substrate miRNAs, and subsequent re-isolation and quantification of imported miRNAs have made this assay system reproducible and free of ER contamination, which previously limited its utility for identifying parameters and factors that control miRNA import into endosomes. For details, consult the reference by Ghosh *et al.* (144). Key Neutral Sphingomyelinase 2 (nSMase2), Vacuolar Protein Sorting-Associated Protein 4 (VPS4), ALIX, ALG-2-interacting protein X; ESCRT, endosomal sorting complexes required for transport; FAN, Factor Associated with Numb; ILV, Intraluminal vesicles.

The RNA-induced silencing complex (RISC), which consists of Argonaute proteins (Ago), plays a significant role in determining the fate of miRNAs in the cytoplasm (123). Ago proteins attach to mature miRNAs, directing them to their target mRNAs to silence genes. The process of incorporating miRNAs into the RISC is tightly controlled by RNA-binding proteins such as TRBP (TAR RNA-binding protein) and PACT (Protein Activator of PKR) (124). These RNA-binding proteins interact with Dicer and Ago2, helping miRNAs form RISCs and enhancing their stability and function in animal cells (124, 125). Ago2 assists in the creation and stabilization of miRNAs and their loading into exosomes and other extracellular vesicles (126). The ESCRT complexes are vital for the creation of various types of EVs, especially exosomes. These complexes aid in the sorting and packaging of different cargoes, including miRNAs, into EVs. Components of the ESCRT machinery interact with adaptor proteins and miRNA-binding proteins to ensure the selective export of miRNAs (127). CD63, a tetraspanin protein commonly found in exosomes, has been implicated in miRNA sorting. It is suggested that CD63 may interact with specific miRNA-protein complexes, aiding in their selective packaging into exosomes (128) (Figs. 2, *A* and *B* and 3*A*).

### RNA export control by hnRNPs

Although how RNAs are loaded into exosomes or EVs is not yet fully understood, a previous study identified sequence motifs in microRNAs that determine their exosomal localization. The protein hnRNPA2B1 binds to these motifs, regulating the loading of miRNAs into exosomes. Sumoylation of hnRNPA2B1 also affects its binding ability. Mutations in these motifs or changes in hnRNPA2B1 levels can impact miRNA packaging. These findings suggest hnRNPA2B1 is crucial for miRNA sorting (129). hnRNPA2B1, a heterogeneous nuclear ribonucleoprotein, recognizes specific miRNA patterns and facilitates their packaging into exosomes. hnRNPA2B1 binds to specific miRNA sequences, called EXOmotifs, which are essential for their selection for exosomal transport (130). This interaction is influenced by sumoylation, a post-translational modification that increases hnRNPA2B1's attraction to miRNAs and their subsequent release. A follow-up work showed that, along with hnRNPA2B1, the proteins Fus, Alyref, and SYNCRIP (Synaptotagmin-binding cytoplasmic RNA-interacting protein) also recognize an array of EXOmotifs other than GGAG and CELLmotifs, for miRNAs exclusively for cellular retention (131) (Figs. 2, *A* and *B*, 3*B* and Table 4).

Mechanistically, hnRNPA2B1 binds to GGAG/UGCA motifs of miR-198 and miR-601 and also interacts with miRNA-17 and miR-93 *via* AGG/UAG motifs. Another RNA-binding protein, heterogeneous nuclear ribonucleoprotein A1 (hnRNPA1), also undergoes sumoylation and binds miR-198, suggesting a potential role in sorting miRNA into EVs (130, 132, 133, 134). On the other hand, depletion of hnRNPA2B1 leads to enhanced levels of miR-503 within EVs, suggesting that hnRNPA2B1 specifically inhibits the loading of miR-503 into EVs (135). Additionally, heterogeneous nuclear ribonucleoprotein C1 (hnRNPC1) has been reported to promote the export of miR-30d *via* EVs during early embryonic development (136).

Another member of this hnRNP family is Synaptotagmin-binding cytoplasmic RNA-interaction protein (SYNCRIP), which also showed direct association with the miRNAs to load them into EVs. SYNCRIP contains an N-terminal unit for RNA recognition (NURR) domain that helps the protein to bind to a GGCU motif of some specific miRNAs, such as miR-3470a and miR-194-2-3p, which are highly enriched in the EV fraction. Deletion of this NURR domain or knockdown of SYNCRIP leads to impaired miRNA binding and, subsequently, reduced enrichment of these proteins in EVs (137, 138). Recent evidence indicates that various protein-miRNA interactions control miRNA compartmentalization, as PCBP2 serves as a key inhibitor of SYNCRIP function in mouse liver cells (139).

Interestingly, the EXOmotif hypothesis for miRNA export regulation cannot fully account for the entire miRNA repertoire in EVs, nor can it explain why some miRNAs with EXOmotif remain inside the cell despite the presence of EXOmotif interactors, possibly due to other miRNA interactors (139). MiRNAs containing CELLmotif are also exported under certain conditions, possibly through other miRNA-recognizing proteins and their partners. Therefore, further refinement of the motifs or structures within miRNAs is necessary, as this process is influenced by several factors, including factors like HuR, which can alternatively affect miRNA fate in response to external cues (Figs. 2*C* and 3*B*).

### RNA export by AU-rich element binding ELAVL proteins

The Embryonic Lethal, Abnormal Vision, Drosophila, Homolog-Like (ELAVL) family protein HuR is the first RNA-binding protein reported to confer reversibility to miRNA-mediated repression in hepatic cells under stress conditions (140). HuR recognizes AU-rich elements on the 3′UTR of mRNAs, stabilizing them and preventing miRNA binding. Interestingly, HuR also plays a crucial role in regulating miRNA levels in hepatic cells during amino acid starvation. HuR relocates from the nucleus and polymerizes on the 3′UTR of target mRNAs, displacing bound miRNPs and derepressing miRNA-bound messages, thereby replacing Ago2-containing miRNPs on the ER (39). The displaced miRNAs bind reversibly to HuR and are subsequently transported to endosome membranes. There, miRNAs are released from HuR due to HuR ubiquitination. Once ubiquitinated, HuR can no longer bind miRNAs due to reduced affinity, thereby facilitating the loading of unbound miRNAs into endosomes (141). Moreover, in different physiological states such as stress or infection, HuR's role in maintaining miRNA levels has been demonstrated. In lipid-loaded liver cells, HuR accelerates the dissociation of Ago2 from Dicer1, promoting Dicer1 export *via* EVs and miRNA export (142). There are also reports on HuR’s role in sorting miRNAs like miR-155 and let-7a into EVs (143).

Recent studies have provided detailed insights into the mechanisms of HuR-mediated miRNA export. Using a new endosome-based reconstitution assay, the role of the endosomal RalA GTPase in the process has been identified. To help load miRNA into MVBs, the HuR-miRNA complex binds to and activates the endosomal RalA GTPase, thereby promoting import *via* its GTPase activity (144). It has also been observed that in activated macrophages, HuR can transfer miRNA to the SNARE (soluble N-ethylmaleimide-sensitive factor-activating protein receptor) protein Syntaxin 5 (STX5), which can act redundantly with HuR and can transfer miRNA to endosomes. STX5-miRNA can be entrapped within the lumen of endosomes to form an MVB with STX5-miRNA complex inside (145) (Figs. 2*C* and 3*B*).

In a recent report, Ray *et al.* have identified the miRNA-binding and export functions of another ELAVL protein, HuD (146). These ELAVL4 proteins, expressed predominantly in neurons, are suggested to play a role in the nervous system’s development, maintenance, and function (147). Loss of HuD causes neuronal loss (148), neuroinflammation (149, 150), and accelerated aging (149). HuD’s involvement in Parkinson’s disease (PD) (151), Alzheimer’s disease (AD) (149), and amyotrophic lateral sclerosis (ALS) (152) has been reported before. It is possible that HuD, by exporting specific miRNAs, controls neuronal fate and suppresses the dominance of HuR, serving as the major miRNA regulator in neurons (146). It is important to elucidate the miRNA regulatory and substrate-specific export functions of other neuronal ELAVLs (ELAVL2 and ELAVL3) to clarify the role of miRNA regulation in neurodegenerative and developmental processes (Fig. 2*C* and 3*B*).

### RNA export regulation by YBX1, La, and other RNA-binding proteins

Further research has identified new RNA-binding proteins that transport a specific subset of miRNAs. Y-Box Binding Protein 1 (YBX1) was found to interact with specific miRNAs and aid in their selective incorporation into EVs (153). This protein functions as a mediator, recognizing and binding miRNAs and guiding their export *via* exosomes. YBX1, which, along with RNA transport, also takes part in mRNA splicing (154). After hypoxia/reoxygenation (H/R) treatment of endothelial progenitor cells (EPC), YBX1 controls the export of miR-133 *via* EVs. Additionally, in HEK293T cells, YBX-1 was able to selectively bind miR-223 with the help of its RNA-binding domain and significantly promoted sorting of miR-223 into the EVs (153, 155). YBX1 is responsible for the specific packaging of miR-223 into exosomes through liquid-liquid phase separation (156). YBX1 condensates specifically recruit miR-223 into exosomes secreted by *ex vivo* cultured cells. Point mutations that prevent YBX1 phase separation reduce their incorporation into biomolecular condensates in cells and disrupt the sorting of miR-223 into exosomes. The formation of biomolecular condensates and the entrapment of miRNAs suggested a possible mechanism for their entry into endosomes (156) (Figs. 2*C* and 3*B*).

Furthermore, alterations in transcriptome dynamics and KRAS activity can affect miRNA sorting into MVBs in various contexts; thus, KRAS is predicted to play a key role in miRNA packaging into exosomes (157). Recent findings indicate that the Lupus La protein has a strong affinity for motifs in miR-122, enabling it to export the miRNA (158). Lupus La protein is an RNA-binding protein associated with RNA Pol III that continuously shuttles between the nucleus and the cytosol. It has a very strong binding affinity for miR-122 and enhances the secretion of this miRNA *via* EVs in triple-negative breast cancer MD-MBA231 cells. Depletion of this protein leads to a significant reduction in the enrichment of miR-122 in the EV fraction (158, 159). A follow-up study reports the importance of p62 in sorting of miR-122-bound La to endosomes and their export. This suggests that p62/ATG7 may affect miRNA sorting into endosomes (160).

The mechanism of selective binding and regulation demonstrates how RNA-binding proteins can modulate miRNA export in response to cellular requirements and external signals. Additionally, miRNA exports are affected by interactions between RNA-binding proteins and cytoskeletal components. The cytoskeleton provides structural support for vesicle transport, with proteins such as actin and tubulin essential for this function (161). Disrupting actin polymerization, for example, has been shown to inhibit exosome release, thereby reducing the export of miRNA and mRNA. This correlation highlights the importance of the dynamic interplay between RNA-binding proteins and the cytoskeletal network in ensuring effective miRNA export.

Syndecans, notably Syndecan-4, along with their associated partner, syntenin, have been linked to EV development and loading. These proteins help select specific miRNAs for inclusion in exosomes, possibly through interactions with the ESCRT system (162). Vaults are multi-subunit structures that may be involved in nuclear-cytoplasmic transport. The Major Vault Protein (MVP), a crucial constituent of vaults, is involved in the selective export of miRNAs, including miR-193a. It has been demonstrated to interact with miRNAs and promote their packaging into exosomes for export (163) (Fig. 3, *C* and *D*).

In addition, the presence of RNA-binding proteins and their interacting partners across various cellular compartments introduces an additional layer of regulation. For example, the localization of RNA-binding proteins such as Ago2 to P-bodies, cytoplasmic granules involved in mRNA turnover, suggests spatial regulation of miRNA sorting and export. The dynamic movement of Ago2 and other RNA-binding proteins between P-bodies and the endosomal pathway underscores the intricate coordination required for miRNA export (164). Furthermore, non-coding RNAs (ncRNAs), including long non-coding RNAs (lncRNAs) and circular RNAs (circRNAs), can serve as sponges or decoys for miRNAs and RNA-binding proteins, influencing their availability and function. For example, lncRNAs such as MALAT1 and circRNAs such as CDR1as have been shown to bind to miRNAs and RNA-binding proteins, sequestering them and altering their activity. This network of interactions adds complexity to the regulation of miRNA export and function (165, 166).

Hence, the process of transporting miRNAs out of the cell is complex and governed by a network of RNA-binding proteins and their associated factors. Essential RNA-binding proteins like Ago2, HuR, and hnRNPA2B1, or YBX1, along with lipid messengers such as ceramide, vesicle transport regulators like Rab GTPases, and the cellular structural framework, work together to ensure the effective export of miRNAs. Disruption of these mechanisms can lead to pathological conditions, underscoring the importance of understanding these processes for potential therapeutic interventions (Fig. 3*C*).

### Involvement of non-RNA binding proteins in miRNA export

As mentioned earlier, several RNA-binding proteins (RNA-binding proteins), including hnRNPA2B1, SYNCRIP, YBX1, MVP, HuR, HuD and La proteins, play key roles in the EV-mediated export of various miRNAs from donor cells. In addition to these RNA-binding proteins, other non-RNA-binding proteins, including membrane proteins, small GTPases, and SNARE proteins, are involved in the export of cellular miRNAs *via* EVs (Fig. 3, *A*–*D* and Table 4).

### Membrane proteins

There are several membrane proteins, including caveolin-1 (Cav-1), Neutral Sphingomyelinase 2 (nSMase2), Vacuolar Protein Sorting-Associated Protein 4 (VPS4), and others, that play a crucial role in EV biogenesis and also in the EV-mediated export of miRNAs. Cav-1 is a membrane protein that resides within the small invaginations of the plasma membrane and regulates vesicular membrane trafficking. Due to stress conditions, including hyperoxia or excessive ROS generation, the cellular Cav-1 level shows an upregulation, and this elevated level of Cav-1 promotes the secretion of hnRNPA2B1 and hnRNPA2B1-associated miRNAs into MVBs for their subsequent export *via* EVs (133). nSMase2 is a hydrolase that regulates ceramide biosynthesis, a process largely involved in EV biogenesis. Inhibition of nSMase2 has been reported to significantly reduce cellular miRNA export, and this nSMase2-mediated sorting of miRNAs into EVs has potential implications for tumor signaling and progression. For example, tumor cells accelerate nSMase2-dependent export of miR-16 to prevent apoptosis and promote cell proliferation (167, 168).

Vacuolar protein sorting-associated protein 4A (VPS4A) is a membrane protein that plays a role in MVB sorting and membrane trafficking. In hepatocellular carcinoma (HCC) cells, overexpressing VPS4A results in increased levels of exosomal cancer-promoting oncomiRs (oncogenic miRNAs) such as miR-27b-3p and miR-92a-3p that act as oncogenes, as well as tumor suppressor miRNAs like miR-193a-3p, miR-320a, and miR-132-3p. These findings indicate that VPS4A fosters a tumor-suppressive intracellular environment while simultaneously enhancing the secretion of oncomiRs *via* EVs, which in turn promote tumor migration in recipient cells (169, 170) (Fig. 3, *C* and *D*).

### Small GTPases

Small GTPases, such as the Ras superfamily and Rab family, are located on endosomal membranes and mainly regulate vesicle trafficking and cargo transport within cells. They may utilize the energy from GTP hydrolysis to enhance miRNA sorting into endosomes, as studies show that a non-hydrolyzable GTP analog (GTPγS) blocks miRNA packaging into endosomes (144, 171, 172). Ras-like small GTPase protein, Ral, has been reported to interact directly with Phospholipase D1 (PLD1), which regulates the vesicular trafficking between the plasma membrane, Golgi apparatus, and endoplasmic reticulum. PLD catalyzes the hydrolysis of phosphatidylcholine (PC) into phosphatidic acid (PA). Ral GTPases control PLD1 localization on MVBs, which is required for local PA accumulation and ultimately for MVB biogenesis and exosome secretion (173). Previous reports also suggest that RalA promotes MVB formation *via* the syntenin-syndecan pathway and enhances exosomal release from cells (174, 175). A recent study has shown that RalA also interacts directly with the miRNA exporter protein HuR to facilitate the miRNA loading into MVBs. Although the exact mechanism remains incompletely understood, the study confirmed that RalA recruits the HuR-miRNA complex, which stimulates its GTPase activity to promote the sorting of miRNA into MVBs (144).

### SNARE proteins

Soluble N-ethylmaleimide-sensitive factor-activating protein receptor (SNARE) proteins are primarily involved in regulating membrane fusion. Specific pairing between vesicle SNARE (v-SNARE) and its cognate target SNARE (t-SNARE) forms the SNARE complex required for fusion of these vesicle membranes (176, 177). Interestingly, in *C. elegans*, despite being a Golgi SNARE, Syntaxin-5 (Syx-5) has been shown to participate in MVB fusion with the plasma membrane, thereby promoting exosome secretion in coordination with the Ral1 small GTPase (174). A recent study reported that the human homolog of syntaxin-5 (STX5) lowers cellular miRNA levels, including miR-122, let-7a, miR-16, miR-21, and miR-155, by facilitating EV-mediated export of miRNAs. STX5 promotes the interaction between the ER and endosomes to facilitate the enrichment of miRNAs into MVBs and then accelerates the fusion between these miRNA-loaded MVBs and the plasma membrane to release the miRNAs in the extracellular milieu *via* EVs. Moreover, it has been observed that STX5 interacts directly with the miRNA uncoupler protein HuR in activated macrophages (145). This study has revealed a *de novo* RNA-binding property of the SNARE protein STX5. HuR uncouples miRNAs from the target miRNP complex and subsequently transfers those miRNAs to the N-terminal disordered region of STX5, and the STX5-miRNA complex gets loaded into the endosomal compartments. Additionally, STX5 promotes fusion of these endosomes with the plasma membrane and inhibits their lysosomal targeting (145). Overall, the emerging potential of recently reported non-classical RNA-binding proteins in EV-mediated miRNA export suggests that several unexplored mechanisms may control the sorting and loading of miRNAs into EVs.

## Other cell intrinsic and extrinsic factors controlling miRNA export

### Membrane dynamics and miRNA export

Cellular miRNA activity and its export depend heavily on intracellular membrane dynamics and vesicular trafficking. Several organellar membranes are involved in distinct miRNA-related events, including miRNA processing, miRNA-mediated gene silencing, and miRNA export *via* EVs. miRNAs are initially expressed as long transcripts in the nucleus and then undergo two rounds of processing to form the mature miRNAs. After the first round of processing, the double-stranded precursor form of miRNAs is exported out of the nucleus to the cytosol by a membrane protein complex, Exportin-5/Ran-GTP(37). In the cytosol, rER-attached polysomes play a crucial role in miRISC-mediated silencing. Reports suggest that miRNA, Ago2, and target mRNA assemble on the surface of rER-attached polysomes before translational repression takes place (178). Subsequently, this miRISC and target mRNAs are targeted to endosomal compartments (43, 179).

Apart from rER, several other organelles participate in this miRISC-mediated silencing process, especially various endosomal vesicles in the endocytic pathway, which are closely involved in the EV-mediated export of miRNAs. Biogenesis of extracellular vesicles largely depends on the cellular endosomal pathway. Initially, the plasma membrane invaginates to release clathrin-coated vesicles inside the cells that turn into the early endosomes, and these early endosomes, upon maturation, form the late endosomes (LE) or multivesicular bodies (MVBs). As the name suggests, MVBs do contain several intraluminal vesicles (ILVs) that are formed from the inward budding of endosomal membranes. Finally, these MVBs fuse with the plasma membrane, releasing their internal ILVs into the extracellular milieu (180, 181). Recent studies have reported that membrane contact sites (MCSs) between the ER and LE significantly regulate extracellular vesicle secretion. ER-LE MCS could also mechanistically impact the endosomal maturation, LE motility, and LE positioning. Moreover, it can affect the final switch of small GTPases to Rab27a, promoting fusion of MVBs with the plasma membrane. Various proteins, including protrudin (important for anterograde transport), Oxysterol-binding protein-related protein 1L (ORP1L; the cholesterol sensor), Lysosomal-associated membrane protein 1 (LAMP1; organizational scaffold protein), and VAP-A (coordinates with ceramide transfer protein CERT), are present at the ER-LE MCS and are also closely involved in LE accumulation, LE translocation to the plasma membrane, and RNA loading into secretory MVBs (182, 183, 184).

Formation of these endosomal compartments, including MVBs and ILVs, depends on the endosomal sorting complex required for transport (ESCRT) family of proteins. ESCRT is composed of approximately 20 proteins categorized into four complexes (ESCRT-0, I, II, and III) and a few associated factors such as ALIX, VPS4, and VTA1 (185, 186). Similar to ILV biogenesis, EV biogenesis can occur in either an ESCRT-dependent or ESCRT-independent manner. HRS, a member of ESCRT-0, recognizes and sequesters ubiquitinated proteins on the endosomal membrane and recruits ESCRT-I and II at the site where they carry out the inward budding of the membrane packed with sorted cargoes, and finally, ESCRT-I and II recruit ESCRT-III with the help of ALG-2-interacting protein X (ALIX) to complete the pinching off of the vesicles to form the ILVs (180, 187) (Figs. 2*A* and 3*A*). In tumor cells, it has been reported that the ESCRT-associated protein ALIX interacts with the syndecan heparan sulfate proteoglycan *via* syntenin to facilitate the formation of ILVs. ALIX-syndecan interaction also drives the packaging of the cargo of interest into the vesicles (162, 188). ESCRT-independent mechanism mainly involves hydrolysis of sphingomyelin to ceramide by type-II sphingomyelinase. Ceramide can associate with other ceramide molecules to form lipid raft-like structures, leading to the initial inward budding of the endosomal membranes to form ILVs (189, 190) (Fig. 3, *C* and *D*).

Motor proteins help mobilize secretory vesicles along with the cellular cytoskeleton. Different Rab proteins are associated with different types of endosomal vesicles. Rab27a and Rab27b are involved in MVB docking and exosomal export (191). Apart from the Rab proteins, Ras-related GTPases homolog, Ral1, also showed enhanced ILV formation and mediates fusion of MVBs to plasma membrane with the help of its downstream effector protein, SYX-5, to facilitate exosome secretion in *C. elegans* (174). The final fusion step between MVB and plasma membrane is mainly governed by SNAREs. Multiple SNARE proteins (both t-SNARE and v-SNARE) assemble together to form the SNARE complex that tethers the MVBs to the plasma membrane and mediates the fusion process to release ILVs in the extracellular milieu (181). Therefore, targeting these membrane dynamics, the endosomal pathway, or the ER-LE MCS could contribute to the development of novel approaches to modulate miRNA export and EV-mediated cell-to-cell communication in disease.

A recent report suggests that lipid droplets serve as storage sites for miRNAs, particularly for storing Ago-uncoupled miRNAs to buffer cellular miRNA levels. When miRNAs associate with LDs, they negatively impact miRNA loading into endosomes, thereby restricting EV-mediated miRNA export. HuR, a protein essential for EV-mediated export of miR-122, also facilitates the uncoupling of Ago2 miRNAs and their storage on LDs (45).

### Effect of chemical modification of substrate miRNA on export

Chemical modifications influence whether a mature miRNA stays inside the cell or is packaged into extracellular vesicles (EVs) for communication between cells. Recent research shows that m6A modification on mature miRNAs acts as a "sorting signal" (9). These methylated miRNAs are identified by the RNA-binding protein hnRNPA2B1, which helps load them into EVs for export (192). Intracellular functional impairment results from m6A-mediated inhibition of Ago2-miRNA interaction. Meanwhile, m6A promotes EV loading by being recognized by the RNA-binding protein hnRNPA2B1. Additionally, the role of EV-miRNA in the recipient cell depends on their demethylation by the Fat mass and obesity-associated protein (FTO) (192). In cells that have stopped growing, adding a poly(A) tail to miRNAs can reduce their export, causing them to stay within the cell. Non-templated uridylation (addition of U residues) can alter miRNA duplexes, potentially affecting their stability and their pathway to export. 3′ Uridylation Confers miRNAs with Non-canonical Target Repertoires affecting their stability and export (193).

### Contribution of other cargo export pathways on miRNA export *via* EV

Other cargo export pathways—particularly proteins, lipids, and various RNA molecules—play a vital role in modulating miRNA export through extracellular vesicles (EVs). They do so by competing for space, affecting packaging machinery, and influencing cargo selectivity. In certain cells, such as activated T cells, 5′ tRNA fragments (tRFs) are exported into EVs more actively than miRNAs. Increased export of tRFs can directly compete with miRNA loading, leading to a lower proportion of miRNA in these vesicles (194). The export of mRNA and circRNAs may help eliminate excess or abnormal RNA, with their levels affecting the overall capacity for miRNA export (195). Ago2, a key player in RNA-induced silencing complexes (RISC), is involved in actively sorting miRNAs into EVs (196).

Overexpressing specific miRNAs often results in increased Ago2-mediated sorting, indicating that protein machinery can become saturated or specifically recruited to favor miRNA loading over other cytoplasmic components. Connexin43 (Cx43), commonly located at the cell surface, can form channels on EVs that bind specific miRNAs (such as miR-133b) and promote their selective incorporation into EVs, linking lipid trafficking with active protein-driven cargo selection (197). Additionally, mTORC1 and mitochondrial activity affect miRNA export; increased metabolic activity (as seen in proliferating cells) boosts mTORC1 activity, enhancing the recruitment of RNA-binding proteins and promoting miRNA export (198). Under stress conditions, such as nutrient deprivation, proteins like HuR shift from regulating target mRNA in P-bodies to facilitating the export of interacting miRNA *via* EVs (164). The effect of target transcripts on miRNA export has also been documented in other contexts (199). Furthermore, the retention of miRNA with polysomes in growth-arrested or retarded cells may influence export pathways by preventing miRNA loading into endosomes (200), contributing to observed enhanced cellular miRNA levels in growth retarded cells. A recent report describes the miRNA export inhibitory role of amyloid proteins in miRNA export. Specifically, Aβ1-42 oligomers enhance P-body targeting of miRNA, preventing its export and intercellular transfer in astroglial cells. This process also lowers miRNA levels available for cytokine repression, leading to impaired miRNA repressive activity. Notably, this defect can be reversed by the mTORC1 activator protein Rheb (201).

### Assay system to study miRNA export process

Using cellular components from animal cells in a cell-free system is a reliable way to explore key biological pathways and study the molecular events and players involved (202). This approach allows researchers to easily monitor and tweak variables, helping us understand how these pathways work. Researchers are actively studying the endocytic pathway, uncovering new factors and their roles through various biological and genetic methods. Until recently, no one had reported developing an *ex vivo* assay system using isolated endosomes to study miRNA trafficking to the endocytic lumen, probably because it's hard to obtain enough endosomes for such experiments.

A recent approach involved enriching endosomes, verifying their purity, and characterizing them with NTA (Nanoparticle Tracking Analysis) and AFM (Atomic Force Microscopy) analyses prior to using the enriched fractions in a test to examine how miRNA enters endosomes (144). This is important given the lack of a reproducible method to have pure endosomes. Other methods for testing miRNA packaging into vesicles often use synthetic lipids to create artificial vesicles or rely on crude membrane preparations (156, 203) that do not exclude non-endosomal cellular components, such as the endoplasmic reticulum, which can introduce substantial noise because ER membranes are rich in miRNAs. This crude membrane fraction-based assay system was used to study La protein-driven entry of miR-122 in TNBC cell line-derived membrane/MVB and to identify the role of p62 in MVB entry of miRNAs (156, 203). Carefully purified endosomes, with no or minimal contamination from the ER and mitochondria, pre-removed by density gradient separation and re-isolation before the assay, are key to reproducible results. The recent assay used the more enriched endosomal fraction to monitor structural changes in vesicles as they matured to larger MVB-like structures after the reaction, using NTA and AFM (Fig. 3*E*), and met the criteria as a primary method for studying miRNA packaging into endosomes, an *in vitro* reconstitution assay with high reproducibility (144).

These assay systems allow us to observe molecular and cellular processes *ex vivo*, where we can closely monitor or control many variables to better understand their roles. However, since cellular reconstitution focuses only on a specific set of components used by cells, it may not always capture the exact mechanisms of the biological process and is somewhat artificial in its mimicry of the *in vivo* situation. Despite this limitation, the potential of this assay system is enormous for identifying key steps and features of miRNA export and turnover *via* the endosome-MVB pathway that can be easily verified in parallel *ex vivo* and *in vivo* assays (204). To bridge the gap between simplified subcellular reconstruction and the complexity of cellular physiology, it's helpful to combine *in vitro* reconstitution and cell-based *in vivo* validation approaches. Studying a cellular phenomenon from both perspectives ultimately will give us a more detailed and complete understanding of miRNA export process.

### Cooperativity in miRNA export

A recent study highlighted cooperativity in miRNA export. Ghosh *et al.*, in their recent work, used *ex vivo* and *in vitro* assays to confirm that the presence of one miRNA can influence the export and cellular levels of other miRNAs in macrophages (204). By examining how hepatic miR-122 alters the miRNA profile of target macrophages, it has been shown that miR-122, in the presence of miRNA-binder ELAVL1 HuR, contributes to the export of other miRNAs, including the anti-inflammatory miR-146a, potentially facilitating macrophage activation by pro-inflammatory miR-122. Cooperative binding of miR-146a with HuR in the presence of miR-122 enhances its endosomal loading and export. This suggests a more robust and cooperative role for RNA-binding proteins, ultimately ensuring context-dependent changes in the miRNA content of released EVs.

## Evolution of the miRNA export process

### miRNA evolution

The evolutionary history of microRNAs (miRNAs) reflects a key transition from an ancient viral defense mechanism to a complex regulatory system that enhances multicellular development (205). MiRNAs likely evolved independently in plants and animals through convergent evolution, sharing core proteins like Dicer and Argonaute but differing in processing, genomic location, and target regulation. Their ancestral machinery originated in prokaryotes and was present in the Last Eukaryotic Common Ancestor, initially functioning as an immune defense using siRNAs against viruses. Over time, lineages repurposed these RNAi pathways to regulate internal genes (205, 206).

The evolutionary paths of plants and animals diverged, with miRNA genes constantly emerging and disappearing through a rapid birth-and-death process, often originating from mutations or transposable elements. Most young miRNAs are weakly expressed and lack targets, but a small fraction acquire beneficial targets, become conserved, and integrate into regulatory networks, contributing to developmental robustness (207). The expansion of miRNAs correlates strongly with the rise of complex body plans, with significant bursts of innovation at key evolutionary milestones, including the origins of bilaterians, vertebrates, and placental mammals. Instead of evolving new proteins, complex organisms expanded their miRNA repertoires to finely tune gene expression, allowing for specialized tissues and organs (208).

### miRNA-transport evolution

The evolution of microRNA (miRNA) transport reflects a shift from basic antiviral defense to intricate intercellular and cross-kingdom communication networks. While the fundamental enzymatic machinery for small RNA processing existed in the last eukaryotic common ancestor (LECA), mechanisms for transporting these signals across membranes and tissue barriers evolved independently and convergently across various kingdoms (209).

Initially, miRNA transport faced the challenge of crossing the nuclear envelope. In early metazoans, the Microprocessor complex (Drosha/DGCR8) facilitated nuclear processing, necessitating Exportin-5 (XPO5) to export pre-miRNAs through nuclear pores into the cytoplasm. Plants, however, developed a different pathway, processing primary and precursor miRNAs entirely within the nucleus *via* DCL1, and exporting mature miRNA duplexes directly to the cytoplasm using HASTY (HST), the plant ortholog of Exportin-5 (210). As multicellularity evolved, organisms required coordinated gene regulation between neighboring cells. Plants and animals developed contrasting structural adaptations for local cell-to-cell miRNA movement: plants use plasmodesmata for direct cytoplasmic transport of naked or protein-bound miRNAs, while animals utilize gap junctions, exocytosis, and endocytosis pathways for short-range transfer.

With increasing organismal complexity, miRNAs gained systemic signaling roles. Plants use their vascular phloem to transport miRNAs over long distances, such as from leaves to roots to regulate nutrient responses. Animals evolved protective packaging for circulating miRNAs, including extracellular vesicles like exosomes and microvesicles, or binding to proteins such as HDL and AGO2, to ensure stability outside cells (205).

The latest evolutionary development involves cross-kingdom communication, where miRNAs are shuttled between species. Plants and animals deploy extracellular vesicle pathways to transfer silencing miRNAs into pathogens or host tissues—for example, Arabidopsis delivers EVs to fungi, and parasitic nematodes release vesicles to suppress immune responses in mammals. Additionally, dietary miRNAs influence development, exemplified by honeybees ingesting plant miRNAs that guide larval differentiation (211, 212, 213). In summary, miRNA transport evolved from intracellular compartmentalization to systemic, protective, and cross-species signaling mechanisms, transforming a cellular defense tool into a crucial component of ecological and physiological communication.

It is notable that vesicle-mediated communication with the external environment is evolutionarily conserved; from prokaryotes to eukaryotes, and vesicle formation and release are important events (214, 215). Expression of key proteins involved in EV biogenesis and release is largely conserved across animal species, suggesting that, as with other miRNA regulatory processes and machinery like miRNA biogenesis, these processes also evolved independently in plants and animals and were subsequently shaped by evolutionary pressure to facilitate selective cargo loading (*i.e.*, miRNA loading) (145, 174, 175). In *C*. *elegans*, the RalA and STX5 homologous proteins were identified as important for exosome release and MBV maturation; in mammalian cells, they were found to be involved in miRNA export, and STX5 was even found to have evolved the ability to bind miRNAs to facilitate their export (145). This ensures the fitness of organisms in a changing environment and under varying growth conditions in unicellular and metazoan animals. Information to date is limited on whether miRNA- and EV-mediated communication is a common mode of intercellular crosstalk across the animal kingdom (216). We posit that this process is governed by the complexity and cargo selectivity of the miRNA export system, which have gradually evolved over time.

How can we recapitulate the evolution of the miRNA export process and study its implications in human cells? One simplistic approach could be to follow EV cargoes from a similar tissue, such as gut mesenchymal cells, macrophages, or liver cells, from different species under identical external or growth conditions (like metabolic stress or LPS-mediated activation) that can be identified across species to assess the conservation of the miRNA cargoes they secrete. It may be combined with systematic proteomic analysis of the respective cells to identify candidate ESCRT factors and RNA-binding proteins that, by interacting with export signals, may affect miRNA export, thereby ensuring cooperativity and selectivity in an evolutionarily conserved manner.

### A unified model of miRNA export: Evolution of passive mono to selective multicomponent system for miRNAs export *via* EVs

Limitations and refinements of the EXOmotif hypothesis are evident, as not all miRNAs exported in small EVs possess recognized EXOmotifs. This suggests that several independent mechanisms are responsible for vesicular encapsulation. The export process is highly dependent on cell health, and stressors such as serum deprivation, hypoxia, or metabolic shifts (*e.g.*, elevated lipid levels) can markedly alter the miRNA export profile, regardless of the intrinsic motifs present in miRNAs. Different cell types prefer distinct sorting sequences and RNA-binding proteins, so no single universal "addressing" system explains the profiles of exported miRNAs.

The roles of other factors and proteins in this process are more prominent. HuR (ELAVL1) plays a crucial role in the selectivity of miRNA export. It can bind to miRNAs and promote their entry into endosomes, especially during cellular stress, thereby enabling the context-dependent release of gene-repressing miRNAs (141). While SYNCRIP (hnRNPQ) has been identified as a component of the hepatocyte exosomal sorting machinery, recent studies suggest that PCBP2 acts antagonistically by recognizing a CELLmotif to facilitate retention, often competing with the export machinery (139). The export of certain miRNAs, such as miR-671-3P and miR-943, is independent of ATP depletion, suggesting that alternative, energy-independent pathways exist, possibly involving different protein partners or transport mechanisms (217). Evidence suggests that specific miRNAs (*e.g.*, miR-122) can act as regulators, facilitating the co-packaging and export of secondary, low-affinity miRNAs, a process that may be further mediated by HuR (204). Thus, further studies are necessary to refine these export-retention motif models to account for the impact of secondary structure, modified nucleosides, and the specific composition of the EV-binding protein complex in response to changing external cues (204), thereby determining a cell’s miRNA export profile.

### How cellular miRNA abundance may affect miRNA export

Cellular miRNA levels directly influence their export *via* EVs, primarily through a passive process in which elevated intracellular concentrations increase export (218, 219). Nonetheless, specialized active sorting mechanisms, often involving RNA-binding proteins such as HuR, YBX1 or La are used for exporting miRNAs based on cellular metabolic states rather than mere abundance (204, 220). This enables cells to selectively export certain miRNAs even when their intracellular levels are relatively low. Proteins such as ALYREF and FUS recognize export motifs, for instance, the EXOmotif on miRNAs, thereby facilitating export regardless of their abundance (131). Conditions such as high cell density or growth arrest may impair miRNA polyadenylation, thereby decreasing export efficiency (200). Conversely, enhanced mTORC1 activity in cancer cells promotes increased export. Certain miRNAs are enriched within EVs despite low cytoplasmic levels, indicating they are primarily transcribed for export purposes (198). However, research indicates that only a small fraction of EVs contain miRNAs, and their impact on recipient cells may be limited, suggesting a focus on selective, functional delivery rather than widespread influence (221).

### Feedforward and regulatory effects of EV-miRNA on recipient and donor cells

How does miRNA signal flow from donor to recipient cells affect the export path? Is there any feedforward effect? Intercellular miRNA trafficking signaling involves intricate feedback and feedforward loops that refine cellular responses (167, 222, 223). The feedforward effect may also be transmitted back to the donor cell *via* EV- and non-EV pathways, thereby balancing miRNA expression in adjacent cells. Hepatic cells with an imbalance in miR-122 expression may be restored to balanced miR-122 expression by reciprocal regulation *via* EV-miR-122 and insulin-like growth factor 1 (IGF1)- mediated feedback, allowing the exchange of miR-122 and anti-miR-122 signals between hepatocytes (224).

Recipient cells rely on miRNAs as essential regulators within sophisticated feedforward loops (FFLs). For example, a primary transcription factor (TF) may regulate a specific miRNA and concurrently target the same protein-coding gene that the miRNA affects. In distinguishing between Coherent and Incoherent loops, a miRNA can serve as a "failsafe," aiding a transcription factor (TF) in repressing a target protein to prevent its expression in particular cell types. Additionally, miRNAs can influence the expression of target genes to keep protein levels within a tightly regulated range despite changes. They can also control their own production or transport mechanisms. For example, miR-182 and miR-183 suppress Argonaute 2 (Ago2), creating a feedback loop that helps maintain stem cell pluripotency (225).

Transferred miRNAs can inhibit positive regulatory motifs in signaling pathways, thereby halting ongoing signaling and enabling rapid, effective adaptation to new extracellular stimuli. However, it remains uncertain how this effect is conveyed back to donor cells. In *Leishmania*-infected macrophages, the internalization of EV-miRNAs is impeded due to compromised mitochondrial function (226). This impairment could cause EVs to accumulate in the extracellular matrix, thereby reducing miRNA secretion from donor cells. As a result, the transfer of inflammatory miRNAs to infected recipient cells is diminished, which may weaken inflammatory signaling in donor cells and help establish an anti-inflammatory environment at the site of infection (226).

### Functional diversity in miRNA export regulatory protein: Causes and consequences

Interestingly, most RNA-binding proteins involved in miRNA export, including HuR, YBX1, La, and hnRNPs, also have multiple RNA regulatory roles distinct from their function in miRNA export. The connection between these dual roles in miRNA export and other steps in gene regulation, like mRNA stabilization, splicing, or translation, remains unclear. It is possible that miRNA transcription, mRNA transcriptional regulation, or mRNA stability are linked to miRNA export. Previous studies have shown that miRNA storage in P-bodies negatively affects EV-mediated miRNA export, and miRNA-target mRNAs also modulate this process (164). Therefore, it is likely that a feedback or feedforward mechanism exists in which miRNA export influences RNA regulatory processes controlled by the same miRNA-binding proteins, which in turn regulate miRNA export.

**Figure 4:**
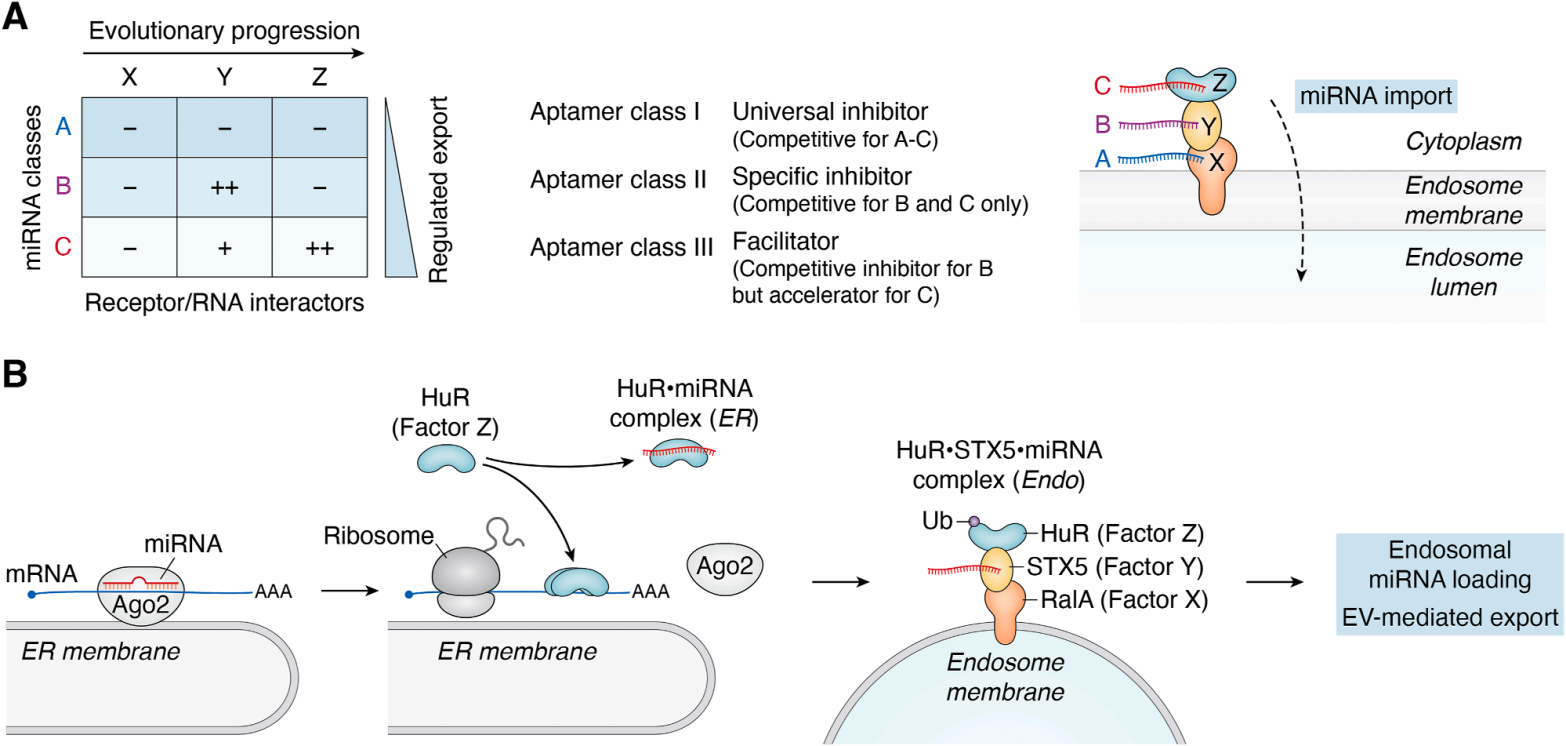
**The possible evolutionary path of three classes of miRNA ligands for endosomal import is defined by their respective interaction partners.***A*, the import matrix illustrates how the absence of proteins X, Y, and Z affects the import levels of three ligand classes: A, B, and C. Factor X is a universal component required for export across all three miRNA classes. The export of class B is more specific than class A and depends on both X and Y proteins. Class C’s export is more tightly regulated, requiring X, Y, Z, and class B RNA for cooperative endosomal import. A simplified model of endosomal import for these miRNA classes is shown, ranging from – to ++, indicating increasing import levels. SELEX-evolved aptamers that represent miRNAs of classes A, B, or C could act as universal inhibitors (class I), specific inhibitors (class II), or could help facilitate miRNA export (for class III). *B*, the import of substrate miRNA into endosomes requires multiple interactions between miRNA and import factors, beginning with polysomes-associated miRNAs. The replacement of miRNAs from Ago2 is facilitated by HuR; Factor Z, which ensures selectivity and cooperativity in transferring the miRNA to STX5; Factor Y, which ensures both the specific entry of miRNAs into endosomes facilitated by the non-specific interactor RalA; Factor X, which does not directly interact with miRNAs.

A potential method to investigate the miRNA export process is to utilize established SELEX (Systematic Evolution of Ligands by Exponential Enrichment) techniques to identify ligands recognized by the endosome-localized miRNA export machinery, in concert with cytosolic factors, to facilitate EV-mediated export. In this approach, the selected ligands will form a group of aptamers with various export signals. We hypothesize that miRNA sequences influence their import specificity and cooperativity. In a hypothetical situation, to identify optimal RNA sequences that improve miRNA loading into endosomes, an RNA SELEX protocol can be employed, using isolated endosomes in an *in vitro* miRNA import assay to select RNA aptamers specifically targeted to endosomes, which are enriched as RNase-protected pools after import. There we may expect three types of miRNA ligands for export *via* EVs in human cells (1): non-specific substrates (Group A) exported by a non-selective mechanism (2), miRNAs requiring specific interactor proteins for export, active under particular conditions such as stress (Group B), and (3) co-exported miRNAs (Group C) that depend on a different set of RNA-interacting proteins (Fig. 4*A*). The first group, exported with core miRNA-export regulatory proteins like RalA, which may also be required by other groups for export, whereas the second group may utilize peripheral proteins like STX5, which are supposedly not involved in the export of non-specific substrates (Group A). Export of group C RNA may involve RNA interactors such as HuR to achieve cooperativity (204). Aptamer RNAs developed through SELEX (Class I, II, and III corresponding to Groups A, B, and C, respectively) will facilitate the classification of miRNAs to study their evolutionary hierarchy. Import assays with these aptamers, performed in systems lacking factors like HuR (Z), STX5 (Y), or RalA (X), will help categorize aptamers as Class A, B, or C (Fig. 4*A*).

Use of aptamers with low import efficiency, along with high-import aptamers or miRNAs such as miR-122 (class B substrate), will identify Class III aptamers/miRNAs. The hypothesis suggests that the co-export mechanism, which may be found in higher eukaryotes [*e.g.*, miR-146a (group C) imported alongside miR-122, a group B miRNA (204)], may have evolved in the latest phase. A comprehensive matrix will be required to map factors (X, Y, Z) to miRNA classes (A, B, C) based on aptamer sequences, associated miRNAs, and their interactions with key export proteins (such as RalA, YBX1, and hnRNPs). These findings should correlate with *in vivo* protein expression data in vertebrate cells, and by analyzing miRNA export profiles of cells depleted of specific interactor proteins, using small RNA sequencing of EVs. By examining interactor protein expression, miRNA export specificity, cooperativity, and context-dependent regulation across different vertebrate cell types, we will better understand the evolution of miRNA export and its drivers (Fig. 4*B*).

### Possible co-evolution of the export machinery of the host and pathogens

Additionally, export mechanisms may have co-evolved in host cells and invading/resident pathogens. Pathogens often hijack host export pathways to release their own components *via* host EVs, thereby aiding infection (227). Conserved cargo selection and biogenesis pathways might facilitate cross-species cargo transfer, crucial for symbiosis and latency during infection (226). Using models such as Leishmania (228, 229) or HIV-1 infection (230, 231) in macrophages or T cells, researchers can analyze how host and pathogen factors are incorporated into EVs from infected cells. It would be valuable to investigate the role of miRNA cargo selectors in loading pathogen-derived RNAs or proteins into EVs. Examining host-derived EVs and their RNA content during infection will reveal interactions between the host and pathogens. It has been shown previously that Leishmania-derived GP63 metalloprotease factors target Dicer1 (232), mTORC1 (198, 233), or HuR (143) to modulate the miRNA machinery and miRNA export. It is likely that cargo selection processes evolve in response to infection, enabling coexistence. Similar mechanisms might apply to other infections in which pathogen-specific factors influence host EV-mediated miRNA export, conferring survival advantages.

The export mechanisms for cargoes released by internalized pathogens are also expected to be co-selected over time. Studying genetic variations in host and pathogen genes involved in EV biogenesis and cargo selection may yield insights, although advanced gene-analysis techniques are required to identify mutations responsible for variability in EV export (234). We suggest that the evolution of the miRNA export machinery in host macrophages is influenced by infection patterns and history. The history of parasite infections increases selection pressures that promote host survival and enhance resistance to infection. Likewise, protozoan exosome export must have been shaped by the prior infection history of the selected host and exhibits co-evolutionary dynamics at the genome level. Studies on host-pathogen interactions are crucial for developing control strategies that target these interconnected hubs, mediated by co-evolved crosstalk between the pathogen and host *via* EVs.

### Advantages of EV-mediated communication of miRNA over protein-ligand membrane receptor-based intercellular communications

**Figure 5:**
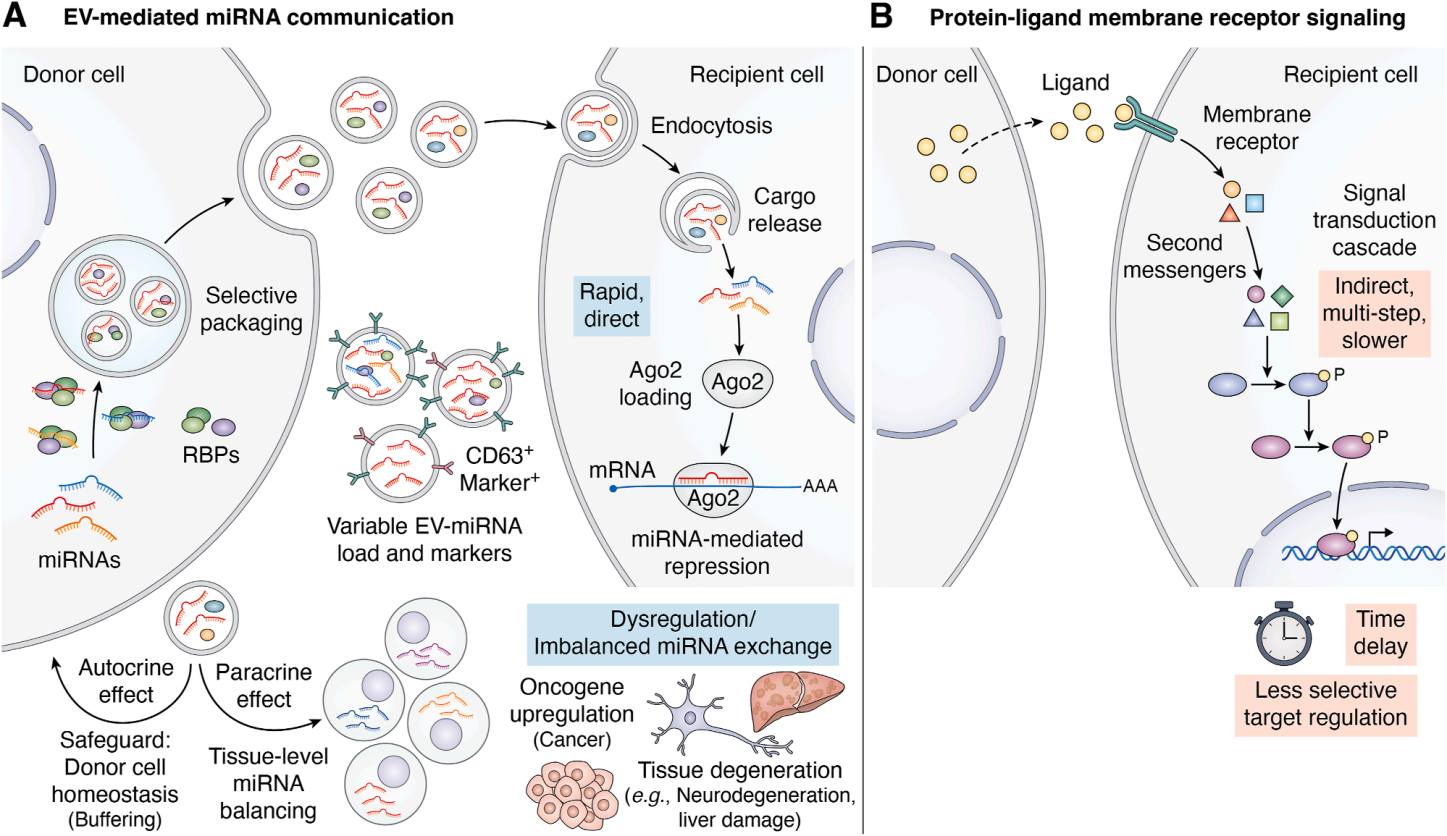
**Comparing two intercellular communication mechanisms: EV-mediated miRNA exchange and classic protein-ligand receptor signaling.***A*, the visual depicts donor cells selectively loading miRNAs into extracellular vesicles *via* RNA-binding proteins; the heterogeneous distribution and selective uptake of EVs by recipient cells (highlighting both autocrine and paracrine effects), and direct miRNA-mediated repression of target mRNAs. This diagram also illustrates EV-miRNA’s role in maintaining cellular and tissue miRNA balance, safeguarding donor cells, and the pathological outcomes of exchange disruption (such as oncogenesis and tissue degeneration). *B*, by contrast, the indirect, slower action of protein-ligand signaling operates on a different timescale. Ensuring regulation at transcriptional and post-transcriptional events.

The misconception that EV-mediated miRNA entry into target cells is insignificant is challenged by the idea of a “vesicular-hormone” role for EV-associated miRNAs. This suggests that EV-miRNAs have autocrine and paracrine functions, with their effects documented to be widespread. However, the average number of miRNA copies per EV is limited, suggesting that miRNAs are heterogeneously distributed among EVs, with some EVs particularly enriched for specific miRNAs. The mechanisms behind this selectivity and enrichment are not yet understood. It is possible that RNA-binding proteins mediate the selective packaging of miRNAs into specific types of EVs, thereby enriching the EVs of interest with specific miRNAs. Moreover, EVs bearing specific surface markers might be selectively internalized and carry specific miRNAs, leading to the significant accumulation of one or more miRNAs in target cells. How the surface marker confers selectivity for internal miRNAs remains an open question and warrants future research, including protocols to isolate individual EVs and to test their effects at the single-cell level. Unlike protein-ligand signaling, miRNAs may have the advantage of being directly internalized *via* endosomal pathways, leading to immediate release, Ago2 loading, and target mRNA repression in recipient cells. This could enable highly selective regulation of specific targets, especially when rapid response is needed—something protein-ligand signaling cannot always achieve (Fig. 5, *A* and *B*). Recent studies on miRNA export cooperativity suggest a robust export process for a subset of miRNAs, resulting in potent effects in recipient cells that are not possible with protein-ligand-based signal transduction (204).

However, miRNA export *via* EVs also serves as a safeguard mechanism for donor cells. By exporting miRNAs, cells buffer their intracellular miRNA levels and regulate miRNA exchange between cells with differing miRNA profiles within tissue, resulting in a 'socialist' activity (235). Interestingly, imbalances in the miRNA repertoire may lead to uneven expression of oncogenes, potentially causing abnormal cell proliferation, exit from the G0 phase, and oncogenic transformation, ultimately leading to cancer. Extracellular vesicle (EV)-mediated microRNA (miRNA) transfer is increasingly identified as a crucial factor in cellular senescence and contributes to age-related functional decline, forming a key part of the "senescence-associated secretory phenotype" (SASP). As tissues age, the molecular cargo within EVs shifts notably, mirroring the stressed, senescent state of donor cells and promoting comparable harmful phenotypes in recipient cells (236). These imbalances contribute to tissue degeneration, such as in the brain and liver, by altering proliferative and degenerative pathways and promoting chronic tissue changes (Fig. 5, *A* and *B*).

### Limitations, caveats, and challenges of EV-mediated miRNA export

Current research indicates that extracellular vesicle (EV) biology is increasingly shifting towards the precise analysis of individual vesicles, despite numerous technical and conceptual challenges (237). The purity of isolated EVs and the scale of engineered EV production pose significant obstacles. Traditional ultracentrifugation (UC) may damage vesicles and co-precipitate contaminants such as lipoproteins and protein aggregates (102, 238). Currently, there is no universally accepted "gold standard" as precipitation methods yield large quantities but low purity, whereas immunoaffinity capture offers high specificity at a higher cost and lower yield (238). Scaling from laboratory to pharmaceutical production remains challenging due to low EV secretion rates by most cells (116). The heterogeneity of isolated EVs presents another substantial challenge, as attributing biological activity to specific subpopulations is currently unfeasible. EVs differ in size, density, lipid content, and cargo, even among those derived from the same cell. Conventional bulk analysis techniques, such as Western blotting or RNA sequencing, may overlook rare but biologically significant subpopulations, underscoring the need for single-particle analysis methods with enhanced capabilities to evaluate the biological activity of isolated EVs.

Linking specific miRNAs to their biogenesis and loading pathways, and distinguishing these steps from EV packaging processes, is difficult due to overlapping markers. The efficiency of cargo loading is low; in some cases, as few as 0.01 copies per vesicle are loaded, rendering the physiological roles of EV-associated miRNAs unclear (239). Differentiating active sorting mechanisms from passive release remains challenging. Many extracellular miRNAs are associated with non-vesicular carriers, such as Argonaute-2 or high-density lipoproteins (HDL), which can lead to an overestimation of EV cargo (139).

The impact of limited critical appraisal on EV research is notably significant. Overinterpretation of data is prevalent, with results from low-resolution techniques often leading to misleading conclusions about EV heterogeneity, size, and composition. Furthermore, issues of reproducibility are compounded by a general lack of standardized, rigorous, and transparent reporting methods, such as those advocated by the Minimal Information for Studies of Extracellular Vesicles (MISEV) guidelines, thereby impeding the ability to compare studies or validate findings. The field is also characterized by a 'hype' culture; the rapid proliferation of interest has sometimes led to overpromising applications lacking sufficient experimental evidence or validation, thereby obscuring the true therapeutic and diagnostic potential of EVs. Conflicting evidence further complicates the landscape, as inadequately defined methodologies produce inconsistent results across laboratories, particularly in cargo identification, owing to variability in extraction, sequencing, and bioinformatics analyses (240).

Challenges persist in subtype analysis and quantification, owing to overlapping physical properties among different EV populations, such as exomeres and various small and large EVs, which hinder accurate distinction and lead to population contamination. Additionally, the absence of specific, exclusive markers for different biogenesis pathways complicates subtype differentiation, despite EVs being highly reflective of their cells of origin. Comparative data remain limited, with a paucity of quantitative studies examining the relative abundance and functional roles of specific EV subpopulations within biological samples (241).

Contamination remains a concern, as conventional isolation methods, such as ultracentrifugation, often fail to effectively separate EVs from other extracellular components, including lipoproteins and protein aggregates, thereby contaminating samples and complicating cargo analysis. To strengthen rigor in EV research, it is essential to adopt single-particle technologies such as nanoflow cytometry and super-resolution microscopy, which improve understanding of heterogeneity and enable precise quantification. The broader implementation of the MISEV guidelines is necessary to standardize methodologies, characterization, and reporting practices, thereby enhancing confidence in research outcomes. Finally, prioritizing functional validation of specific EV subtypes over bulk populations is essential for overcoming current limitations and advancing the field (242).

Emerging Technologies and Perspectives include tools such as Digital Single-EV Analysis (dSEA), which quantifies cargo and markers at the single-particle level using nanofluidics or high-resolution flow cytometry (243). Vesicle heterogeneity is addressed through Tangential Flow Filtration (TFF), a gentle and scalable process that maintains sample integrity during processing of large volumes (244). For purification and scalability purposes, Artificial Nanovesicles (ANVs), synthetically engineered vesicles, serve as reference standards for the behavior of native extracellular vesicles (EVs) (245). Standardization is accomplished using GlyExo-Capture, a methodology that employs glycan-binding magnetic beads to rapidly isolate specific subsets with high purity and efficiency (246). Recent advances in multifactor authentication (MFA) for EV analysis will enable more rigorous, authentic analysis of heterogeneous EV populations, helping to obtain reliable data important for clinical settings (247). Table 5 presents a comprehensive overview of the challenges the field is facing, which are delaying the development of therapeutic EVs.

**Table 5:** Challenges in the miRNA extracellular export research field

Challenge area	Specific knowledge gap & mechanism	Methodological/Biological impact
Selective packaging	Exact mechanisms behind sorting specific miRNAs into vesicles *versus* those retained in cells.	Makes it hard to predict which intracellular miRNAs become circulating biomarkers.
Export triggers	Unknown signaling pathways trigger specific miRNA release during cellular stress (*e.g.*, hypoxia, nutrient starvation).	Prevents standardization of *in vitro* cell culture experiments, leading to data heterogeneity.
Carrier heterogeneity	Distinction between active vesicular transport (exosomes, microvesicles) and non-vesicular transport (*e.g.*, Ago2-miRNA and HDL-miRNA complexes).	High risk of contamination in biofluid samples; makes isolation of pure EV populations difficult.
Energy dynamics	Exact role of cellular ATP and universal *versus* branched transport pathways in outward budding.	Creates conflicting evidence on whether miRNA export is an active, energy-dependent process.
RNA-binding proteins	RNA-binding proteins that mediate miRNA sorting into vesicles, beyond known candidates like Lupus La and HuR.	Limits our understanding of "EXOmotif" or other RNA motif recognition and how RNA-encoded messages are regulated
Recipient cell uptake	Unidentified uptake mechanisms and membrane receptors for specific non-vesicular (*e.g.*, HDL) and vesicular miRNA carriers.	Impairs efforts to study the functional, paracrine effects of cell-to-cell communication *in vivo.*
Standardization	Inconsistent cohorts, technical variations in sample preparation, and a lack of standardized EV isolation protocols.	Prohibits the reproducibility needed to translate extracellular miRNA findings into clinical practice.

One important caveat challenging the concept of miRNA-mediated intercellular communication is the limited number of miRNAs that individual EVs can carry to target cells. Most studies, whether using body fluids or cell culture supernatants, report that miRNA content in EVs is quite low, typically ranging from 1 to 4 miRNA molecules per vesicle (248). When calculated, this amount may not be sufficient to induce significant changes in target or recipient cells (249). However, a prevailing idea is that EV heterogeneity may play a role. A subset of EVs with high miRNA content might be selectively taken up by specific cells, possibly through receptor-ligand interactions between EV-attached ligands and cell-specific receptors (250). This would make EV transfer both selective and efficient, delivering enough miRNA to exert regulatory effects, as supported by numerous studies highlighting the role of EVs in miRNA transfer and their clinical significance.

Considering EV-mediated miRNA transfer as a unidirectional mechanism with relatively high miRNA stability in target cells, it is plausible that continuous, gradual transfer of miRNAs from donor to recipient cells could slowly alter gene expression patterns over days or even years. We hypothesize that dysfunctions related to miRNA transfer or exchange, which become more prominent with age, may influence disease development over a long range of time. Extracellular vesicles (EVs) act as critical mediators of intercellular communication by transferring functional miRNAs between cells. This dynamic transfer shapes age-related physiological changes and systemic genetic imprinting, effectively transmitting a history of cellular interactions and microenvironmental exposures (251). Because EVs establish extensive communication networks, a recipient cell doesn't just receive passive data; it integrates the accumulated "history" of genetic exchanges from its donor cells. This creates a lasting epigenetic imprint that influences how the recipient cell behaves, proliferates, or ages (252).

Alternatively, non-canonical pathways that ensure recipient miRNA loading onto endosome-attached miRNA-free Ago to form miRNPs in target cells, which may interact with their target mRNAs more robustly than regular pre-existing miRNP-mRNA interactions, allowing EV-transferred miRNAs to have higher specific activity than the regular miRNA present. This theory needs experimental validation.

### miRNA export diversity and related mechanisms: Why the differences?

**Figure 6:**
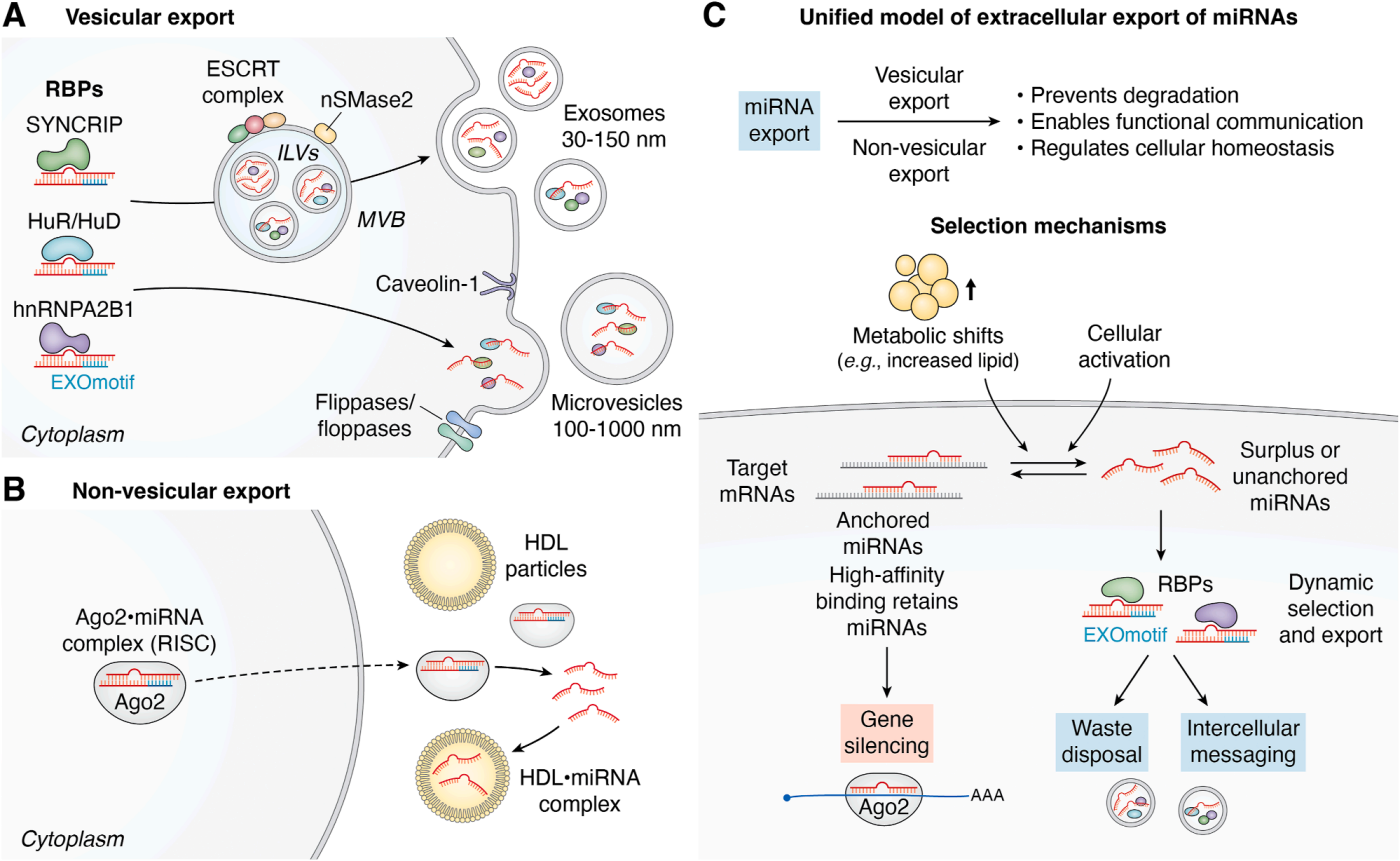
**The unified model of extracellular export of miRNAs.** It explains how microRNAs (miRNAs) avoid degradation and remain functional outside cells *via* a coordinated network of vesicular (*A*) and non-vesicular (*B*) pathways. Vesicular pathways include exosomal biogenesis, in which miRNAs are packaged into exosomes *via* ESCRT and RBPs that recognize EXOmotifs, and microvesicle formation *via* plasma membrane budding regulated by Caveolin-1. Non-vesicular export involves miRNAs bound to proteins like AGO2 or HDL particles, which circulate independently. (*C*) The choice of export depends on molecular factors such as target mRNAs and RBPs, with excess or unanchored miRNAs being actively packaged for disposal or signaling, balancing intracellular retention and extracellular communication.

The unified model of extracellular export of microRNAs (miRNAs) encompasses multiple pathways that elucidate how these small RNAs evade cellular degradation, thereby ensuring their stability and functionality outside the originating cell. Rather than relying on a single mechanism, mammalian cells employ an integrated network comprising vesicular carriers and non-vesicular ribonucleoprotein (RNP) complexes. This network facilitates active intercellular communication, functional pooling, and the disposal of cellular waste. We have proposed the unified model in Figure 6.

### Active vesicular export pathways

Vesicular export involves encapsulating miRNAs within protective lipid bilayers, enabling targeted delivery and uptake by neighboring or distant cells. *Export through the Exosomal Pathway (Endosomal Route) is* initiated with the formation of intraluminal vesicles (ILVs) within Multivesicular Bodies (MVBs). The sorting process is driven by the Endosomal Sorting Complex Required for Transport (ESCRT) and lipid-dependent factors such as neutral sphingomyelinase 2 (nSMase2). RNA-binding proteins (RBPs) identify specific sequences known as EXOmotifs, with proteins like hnRNPA2B1, SYNCRIP, or HuR (or HuD) directing microRNAs (miRNAs) into vesicles, and as mentioned earlier, MVBs subsequently fuse with the plasma membrane, resulting in the release of mature exosomes (30–150 nm) into the extracellular space (Figure 1, Figure 2, Figure 3 and Table 1, Table 2, Table 3, Table 4).

### Microvesicle pathway (Direct budding)

Microvesicles are formed through direct outward shedding and fission of the plasma membrane. This process is regulated by Caveolin-1 and lipid asymmetry changes driven by flippases and floppases. These larger vesicles (100–1000 nm) contain fully processed, single-stranded miRNAs (Fig. 1 and Table 1).

### Non-vesicular protein complex export

Interestingly, most circulating miRNAs (up to 95% or more in some biofluids) are transported independently of vesicles, shielded by specialized protein chaperones. Argonaute 2 (AGO2) proteins interact directly with miRNAs within the RNA-induced silencing complex (RISC), and these AGO2-miRNA complexes can be released or leak outside the cell, maintaining their silencing function. High-density lipoproteins (HDLs) also carry a distinct subset of miRNAs *via* electrostatic interactions, serving as systemic transport carriers that circulate these molecules throughout the body (Table 1 and Fig. 6).

### The structural mechanics of selection

The decision of whether a miRNA remains inside the cell or is exported is highly dynamic and depends on competing molecular factors within the cytoplasm. The cytoplasmic pool of mature miRNA can interact with target mRNAs, which, when bound with high affinity, retain the miRNA for gene silencing. Alternatively, RBPs recognize EXOmotifs, directing miRNAs into vesicles or exosomes. Additionally, miRNAs associated with HDL particles or AGO2 proteins are secreted *via* non-vesicular pathways (Figure 1, Figure 2, Figure 3 and 6 and Tables 1, 3, and 4).

The presence of abundant target mRNAs acts as an intracellular anchor, binding tightly to specific miRNAs and preventing their export. Conversely, when the cell has surplus or unanchored miRNAs, or during metabolic shifts—such as increased lipid levels or cellular activation—active sorting RBPs trigger immediate packaging into vesicles. This process serves a dual purpose: eliminating excess regulatory molecules and functioning as a system for local or systemic messaging.

Thus, overall, the extracellular export process involves multiple stakeholders coordinating among themselves to ensure timely and quantitative export. Nonetheless, there are still issues to be addressed, such as the criteria for cargo selection within each pathway, the number of miRNAs loaded into individual vehicles, and the factors that determine stoichiometry. Additionally, the copy number per cell remains a critical concern, as it is essential to develop methods for incorporating supplementary miRNAs into vesicles in a functional manner.

## Conclusion

While we've made some progress in understanding the main players involved in miRNA export, there's still a lot to learn. We need to explore several aspects in greater depth before we can confidently manipulate miRNA export in mammalian cells for therapeutic benefit. This is especially important when it comes to managing inflammatory responses, like those seen in neurodegeneration, where amyloid proteins have been found to interfere with EV-mediated miRNA export. This process can be rescued by the mTORC1 activator Rheb, suggesting a potential role for restoring miRNA export in combating neurodegeneration (201). Viral proteins like Tat and viral RNA TAR, encoded by the viral genome, may influence miRNA cargo and be released to communicate with other infected tissue cells, including neurons (231, 253). Therefore, preventing the harmful effects of viral proteins and RNA on non-infected cells could involve targeting viral RNA export.

Designing engineered EVs with specific miRNAs is an intriguing idea, and identifying key factors and mechanisms of miRNA entry into EVs could be achieved through new strategies. This is especially relevant in cancer, where exporting specific miRNAs, such as miR-122 in TNBC cells, may be an effective approach to overcoming drug resistance or preventing propagation and metastasis. The development of methods to isolate and monitor EV cargoes at the single-particle level will be crucial in addressing questions about heterogeneity in EV populations, including whether all EVs carry a single or multiple miRNAs. If EVs selectively load specific miRNAs, understanding this selectivity will be an important area of research to enrich the EV population for specific miRNAs.

Does the miRNA turnover mechanism have any crosstalk or reverse regulation with the miRNA export path? The miRNA regulatory pathway involves 'trigger’ RNAs that reverse the usual logic, downregulating miRNAs (254). This target-directed miRNA degradation (TDMD) likely requires a cullin–RING E3 ligase, relying on CUL3 and ubiquitylation factors such as the BC-box protein ZSWIM8 (255, 256). ZSWIM8 is vital for mouse survival during the perinatal period and destabilizes many short-lived miRNAs, highlighting TDMD's biological importance. Biochemical and cellular assays show that AGO binding and polyubiquitylation by the ZSWIM8–CUL3 E3 ligase are key in TDMD, defining a unique cullin–RING E3 ligase class (257). It would be interesting to identify a connection between TDMD and miRNA export, and to understand how the decision to export miRNAs rather than degrade them is ensured.

### Data availability

This manuscript does not contain any new data.

## Conflict of interest

The authors declare that they have no conflicts of interest with the contents of this article.
